# Catalyst-free and solvent-free Michael addition of 1,3-dicarbonyl compounds to nitroalkenes by a grinding method

**DOI:** 10.3762/bjoc.8.61

**Published:** 2012-04-11

**Authors:** Zong-Bo Xie, Na Wang, Ming-Yu Wu, Ting He, Zhang-Gao Le, Xiao-Qi Yu

**Affiliations:** 1 Key Laboratory of Green Chemistry and Technology, Ministry of Education, College of Chemistry, Sichuan University, Chengdu, China, 610064; Fax: (+86) 28-85415886; Tel: (+86) 28-85415886; 2 School of Chemistry Biology and Material Science, East China Institute of Technology, Fuzhou, China, 344000; Fax: (+86)794-8258320; Tel:(+86)794-8258320

**Keywords:** catalyst-free, grinding, Michael addition, solvent-free

## Abstract

An environmentally benign, fast and convenient protocol has been developed for the Michael addition of 1,3-dicarbonyl compounds to β-nitroalkenes in good to excellent yields by a grinding method under catalyst- and solvent-free conditions.

## Introduction

Nowadays, chemists are vigorously taking on the challenge of developing green synthetic methodologies to meet the criteria of sustainable, environmentally conscious development. As a result, catalyst- and solvent-free synthetic methods have attracted much interest not only for laboratory synthesis but also in chemical industry, because of reduced pollution, lower costs, mild conditions, and ease of purification. Recently, practical procedures in the absence of solvents and catalysts have been accomplished for greener and cleaner syntheses [1–6]. As the typical representative of solvent-free reactions, the grinding technique has been widely used in organic synthesis [7–13]. Compared to traditional methods, some organic reactions occur more efficiently in the solid state than in solution due to a more tight and regular arrangement of the substrate molecules [14]. Thus, the grinding mode for solid-state reactions had been applied in the Reformatsky reaction [15], Dieckmann condensation [16], Knoevenagel condensation [17], Aldol condensation [18], etc. [1–2,19–20].

The Michael addition is one of the most fundamental and important reactions for the formation of carbon–carbon bonds and carbon–heteroatom bonds in organic synthesis. The conjugate Michael addition of carbon nucleophiles to electron deficient nitroalkenes is particularly interesting and challenging as it involves the generation of a wide range of different functionalized products from Michael adducts [21–24]. In general, Michael addition reactions require basic or acidic catalysts in organic solvents, as well as long reaction times, which may lead to environmentally hazardous residues and undesirable byproducts [25–30].

As one part of our continuing efforts toward the development of green synthesis methods for Michael additions of nitroalkenes, we have previously reported an enzymatic tandem reaction to form 5-hydroxyimino-4,5-dihydrofurans [22], a transition-metal-free process for the synthesis of substituted dihydrofurans [23] and a catalyst-free tandem reaction for the synthesis of 5-hydroxy-1,5-dihydro-2*H*-pyrrol-2-ones in aqueous medium [24]. Recently, when carrying out the reaction of β-nitrostyrene with 1,3-cyclopentanedione under catalyst- and solvent-free conditions, we were surprised to find that the grinding mode could efficiently promote the reaction, and the corresponding Michael addition product **3a** was obtained in nearly 100% yield. Therefore, we were encouraged to research the Michael addition systematically by the grinding method.

**Scheme 1:**
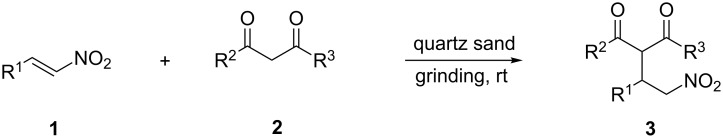
Michael addition under catalyst- and solvent-free conditions.

Herein, we report a green protocol for the Michael addition of 1,3-dicarbonyl compounds to nitroalkenes under catalyst- and solvent-free conditions (Scheme 1). Utilizing this simple, rapid, low-cost and effective procedure, various nitro diketone derivatives were synthesized in high yields.

## Results and Discussion

**Figure 1:**
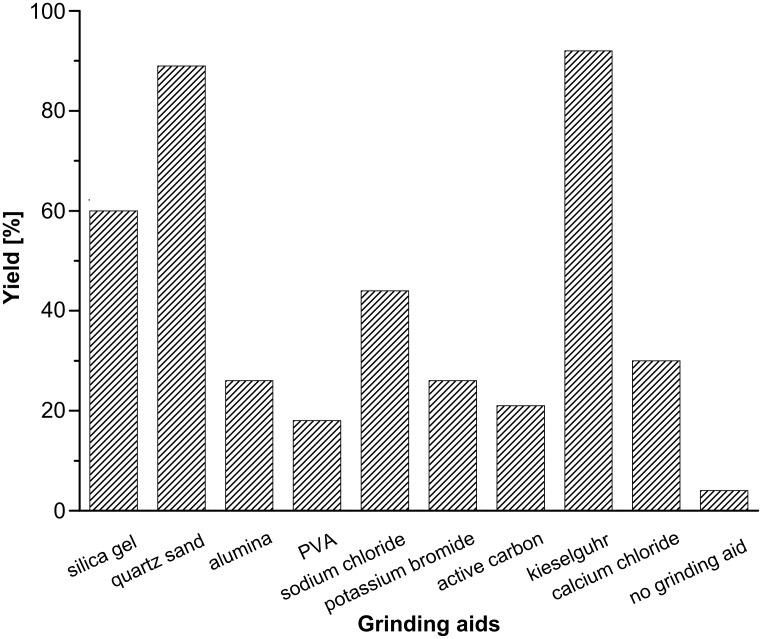
The grinding effect of different grinding aids. Conditions: β-nitrostyrene (14.9 mg, 0.1 mmol), 1,3-cyclopentanedione (9.8 mg, 0.1 mmol), grinding aid (0.50 g), ground for 10 min and then allowed to stand for a further 10 min. Yields were determined by HPLC.

**Figure 2:**
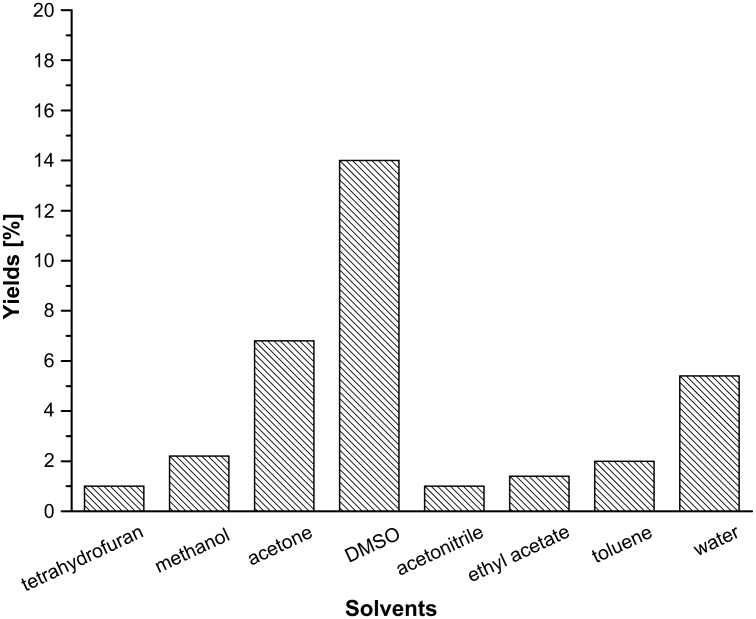
Yields of the model reaction in different solvents. Conditions: β-nitrostyrene (14.9 mg, 0.1 mmol), 1,3-cyclopentanedione (9.8 mg, 0.1 mmol), solvent 1.0 mL, magnetically stirred for 1 h at rt. Yields were determined by HPLC.

In our initial study, equimolar amounts of β-nitrostyrene (**1a**) and 1,3-cyclopentanedione (**2a**), as a model reaction (Table 1, entry 1), were mixed and ground in a mortar at room temperature. The mixture became sticky and adhered to the wall of the mortar firmly after a few seconds, which prevented the reactants from mixing thoroughly and coming into sufficient contact. As a result, only a little of the desired product **3a** was detected, as monitored by thin-layer chromatography (TLC) after 20 min. In an attempt to improve the grinding process, some silica gel was added in the mortar. Surprisingly, a great quantity of Michael product **3a** was obtained after 5 min of grinding. Then a number of powdered substances were screened, such as KBr, quartz sand, Al_2_O_3_, kieselguhr, active carbon, and so on. From Figure 1, it was found that all tested grinding aids could promote the reaction to different degrees, and the primary reason may be that the two reactants could come into contact more effectively after dispersion by the grinding aids. It was found that kieselguhr was slightly better than quartz sand in terms of grinding efficiency, and both gave excellent yields. Although kieselguhr is often used as a catalyst in many organic reactions, quartz sand was selected as the suitable grinding aid owing to its low cost and inertness. To the best of our knowledge, this is the first example in which quartz sand has been successfully used as a grinding aid in Michael addition reactions. Next, the same reaction was performed in several organic solvents, as well as in water in the absence of catalysts for the purpose of demonstrating the high efficiency of the grinding method. As shown in Figure 2, after 1 h of magnetic stirring, the best yield of 14% was achieved in polar DMSO, while the other tested solvents gave much lower yields. In fact, there are few reports on the Michael addition of β-nitrostyrene and 1,3-cyclopentanedione. Hrnčiar and Čulák performed the same reaction in methanol using sodium methylate as a catalyst; however, only 85% of product **3a** was obtained, and a longer reaction time was required [31].

**Figure 3:**
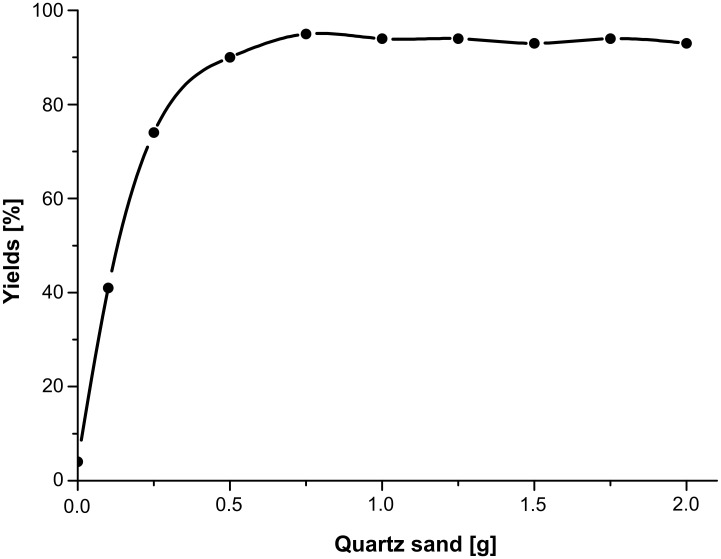
The effect of the amount of quartz sand on the yield. Conditions: β-nitrostyrene (14.9 mg, 0.1 mmol), 1,3-cyclopentanedione (9.8 mg, 0.1 mmol), ground for 10 min and then allowed to stand for a further 10 min. Yields were determined by HPLC.

Subsequently, the amount of quartz sand required was investigated to find the optimal amount on a 0.1 mmol scale (Figure 3). The results showed that 0.75 g or more quartz sand was required for an excellent yield. Generally, the reaction rate decreased with the reduction of the reactant concentration when excessive quartz sand was used. However, the yield did not reduce obviously, even when 2.00 g quartz sand was added in the reaction system. Finally, 0.75 g quartz sand was selected as the grinding aid for 0.1 mmol substrates. To optimize the experimental conditions further, we also examined the effects of the molar ratio of reactants on the yield, and a slightly better result was obtained when 1.2 equiv of nitrostyrene was adopted (data not shown). Also considering that nitroalkenes might polymerize by themselves during the grinding process, a 1.2:1 (acceptor/donor) was chosen as the optimal molar ratio. Having established the optimum conditions, the template reaction was enlarged to a gram scale, and a similar result was obtained (data not shown).

To test the generality of this grinding Michael addition with respect to reactants, different aromatic and heteroaromatic nitroalkenes were used as the acceptors to react with 1,3-dicarbonyl compounds under the optimized conditions. The results are given in Table 1. It can be seen that a wide range of substrates were able to participate in the reaction. A series of substituted β-nitrostyrenes with electron-withdrawing or electron-donating functionalities reacted with 1,3-cyclopentanedione (**2a**) in good to excellent yields. Similarly, the scope of the donor was expanded to other 1,3-dicarbonyl compounds. The best yield (>99%) was obtained for the reaction of furan-2,4(3*H*,5*H*)-dione (**2b**) with β-nitrostyrene (**1a**) (Table 1, entry 14). Some aromatic 1,3-dicarbonyl compounds were also successfully used as donors in this Michael reaction in moderate to good yields (Table 1, entries 18–21). In addition, furylnitrostyrene (**1m**) reacted with 1,3-cyclopentanedione (**2a**) as well as furan-2,4(3*H*,5*H*)-dione (**2b**) to give the corresponding products in excellent or fair yields (Table 1, entries 13 and 22). But no corresponding products were detected in the reactions of β-nitrostyrene (**1a**) with 2-methylcyclopentane-1,3-dione (**2c**) and pyrrolidine-2,5-dione (**2d**) (Table 1, entries 15 and 16). It is noteworthy that the reaction between β-nitrostyrene (**1a**) and cyclohexane-1,3-dione (**2e**) only gave another product through a tandem process (Table 1, entry 17; Supporting Information File 1, Scheme S1). To our delight, Michael products **3n** and **3s** were formed with excellent diastereoselectivity (dr > 99:1, Table 1, entries 14 and 22), product **3l** with moderate diastereoselectivity (dr = 82:18, Table 1, entry 12 ). For the purpose of comparing the reactivity of different nitroalkenes with 1,3-cyclopentanedione, another group of experiments was performed, and a marked difference existed among various nitroalkenes, owing to steric effects or electronic effects (Supporting Information File 1, Table S1).

**Table 1:** Investigation of the reactant scope in the grinding Michael addition.^a^

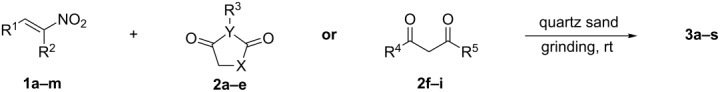					

Entry	Acceptor, **1**	Donor, **2**	Product, **3**	Yield [%]	dr^b^

1	**1a**: R^1^ = Ph, R^2^ = H	**2a**: X = CH_2_, Y = CH, R^3^ = H	**3a**	99^c^	–
2	**1b**: R^1^ = 4-FC_6_H_4_, R^2^ = H	**2a**	**3b**	>99^c^	–
3	**1c**: R^1^ = 4-CF_3_C_6_H_4_, R^2^ = H	**2a**	**3c**	>99^c^	–
4	**1d**: R^1^ = 4-ClC_6_H_4_, R^2^ = H	**2a**	**3d**	>99^c^	–
5	**1e**: R^1^ = 3-ClC_6_H_4_, R^2^ = H	**2a**	**3e**	>99^c^	–
6	**1f**: R^1^ = 4-BrC_6_H_4_, R^2^ = H	**2a**	**3f**	99^c^	–
7	**1g**: R^1^ = 2-BrC_6_H_4_, R^2^ = H	**2a**	**3g**	>99^c^	–
8	**1h**: R^1^ = 4-NO_2_C_6_H_4_, R^2^ = H	**2a**	**3h**	99^c^	–
9	**1i**: R^1^ = 2-NO_2_C_6_H_4_, R^2^ = H	**2a**	**3i**	91^d^	–
10	**1j**: R^1^ = 4-MeC_6_H_4_, R^2^ = H	**2a**	**3j**	>99^c^	–
11	**1k**: R^1^ = 4-MeOC_6_H_4_, R^2^ = H	**2a**	**3k**	>99^c^	–
12	**1l**: R^1^ = Ph, R^2^ = Me	**2a**	**3l**	83^d^	82:18
13	**1m**: R^1^ = 2-furanyl, R^2^ = H	**2a**	**3m**	>99^c^	–
14	**1a**	**2b**: X = O, Y = CH, R^3^ = H	**3n**	>99^c^	>99:1
15	**1a**	**2c**: X = CH_2_, Y = CH, R^3^ = Me	–	n.d.^e^	–
16	**1a**	**2d**: X =CH_2_, Y = N, R^3^ = H	–	n.d.^e^	–
17	**1a**	**2e**: X = CH_2_CH_2_, Y = CH, R^3^ = H	–	n.d.^f^	–
18	**1a**	**2f**: R^4^ = Ph, R^5^ = Me	**3o**	70^d^	53:47
19	**1a**	**2g**: R^4^ = Ph, R^5^ = OEt	**3p**	81^d^	55:45
20	**1a**	**2h**: R^4^ = 4-MeOC_6_H_4_, R^5^ = OEt	**3q**	66^d^	59:41
21	**1a**	**2i**: R^4^ = R^5^ = Ph	**3r**	58^d^	–
22	**1m**	**2b**	**3s**	63^d^	>99:1

^a^Conditions: acceptor (0.36 mmol), donor (0.30 mmol), quartz sand (2.25 g), ground occasionally at room temperature. Reaction time: the exact reaction time was not determined owing to the discontinuous grinding process, but most reactions were complete in 3 h. However, a longer time was required for some reactions (e.g., entries 9 and 12), and good yields were obtained after overnight standing. ^b^dr was determined by ^1^H NMR. ^c^Yields of the isolated product after elution from a sand core funnel. ^d^Yields of the isolated product after chromatography on silica gel. ^e^n.d. = not detected. ^f^No Michael product was detected, and another product was only obtained by tandem coupling.

After completion of the reaction, the mixture was purified directly by flash column chromatography to give the product without the need for any pretreatment. However, a more efficient and convenient purification procedure was developed in our research lab in order to meet the requirement of green chemistry. Namely, the reaction mixture was filtered through a sand core funnel containing a thin layer of silica gel and the pure product was obtained. More than half of the products could be rapidly purified in this way with similar or higher yields and only a little eluent was needed.

## Conclusion

In summary, a convenient, efficient and rapid method was developed for the Michael addition of 1,3-dicarbonyl compounds to β-nitroalkenes in good to excellent yields by a grinding method under catalyst- and solvent-free conditions. It was more meaningful to find that quartz sand could effectively promote this reaction by acting as a grinding aid. The reactions could be performed smoothly between solid–solid or solid–liquid materials at room temperature with a wide range of reactants. Moreover, a much simpler purification procedure was developed, in place of column chromatography.

## Supporting Information

File 1 General procedures and analytical data.
