# Supplementary material for: Catalyst-free and solvent-free Michael addition of 1,3-dicarbonyl compounds to nitroalkenes by a grinding method
Source: Beilstein J Org Chem. 2012 Apr 11;8:534–8. doi: 10.3762/bjoc.8.61 (PMC3343280; doi:10.3762/bjoc.8.61)
Supplement: File 1 — General procedures and analytical data. [file Beilstein_J_Org_Chem-08-534-s001.pdf]

Supporting Information
for
Catalyst-free and solvent-free Michael addition of 1,3-dicarbonyl compounds to nitroalkenes by a grinding method

Zong-Bo Xie[1,2], Na Wang*[1], Ming-Yu Wu[1], Ting He[1], Zhang-Gao Le[2] and Xiao-Qi Yu*[1]

Address: [1]Key Laboratory of Green Chemistry and Technology, Ministry of Education, College of Chemistry, Sichuan University, Chengdu, China, 610064; Fax: (+86) 28-85415886; Tel: (+86) 28-85415886 and [2]School of Chemistry Biology and Material Science, East China Institute of Technology, Fuzhou, China, 344000; Fax: (+86)794-8258320; Tel:(+86)794-8258320

E-mail: Xiao-Qi Yu - xqyu@scu.edu.cn

*Corresponding author

General procedures and analytical data

Materials and general information

All reagents were obtained from commercial suppliers and were used without further purification unless otherwise noted. 1,3-cyclopentanedione was purified by flash column chromatography (silicagel, 300–400 mesh) prior to use. ^{1}H NMR and ^{13}C NMR spectra were recorded on a Bruker 400 MHz instrument by using $CDCl_3$ or DMSO-d_6 as a solvent with tetramethylsilane (TMS) as an internal standard at room temperature. Chemical shifts are given in δ relative to TMS; the coupling constants *J* are given in Hz. HPLC was carried out by using a Shimadzu organizer consisting of a LC-2010A HT Integrator and a UV–vis detector. C18 column (150 mm × 46 mm) was used in the HPLC experiments with MeOH/H_2O 30:70, 0.5 mL/min and λ_{max} = 265 nm. HRMS were performed on Bruker Daltonics Bio TOF mass spectrometer. Column chromatography was performed on EM silica gel (200–300 mesh).

General procedure for the preparation of nitroalkenes

Aldehyde (0.05 mol), $MeNO_2$ (0.05 mol), and MeOH (10–20 mL) were added to a round-bottom flask and then stirred vigorously. NaOH solution (10.5 M, 10 mL) was added dropwise in an ice bath; a large amount of white or yellow solid precipitated, and stirring was continued for 15 min. Distilled H_2O was added until the solution became clear, then the solution was added dropwise to concentrated HCl (30 mL), and a yellow solid precipitated. The yellow solid was filtered and washed with H_2O, then evaporated in a vacuum drying oven. After recrystallization (EtOH), yellow needle-like crystals were obtained.

General procedure for the model reaction (optimizing conditions)

A quantity of β-nitrostyrene, 1,3-cyclopentanedione and grinding aid were added in a mortar, and after 10 min of continuous grinding, the mixture was left standing at rt for 10 min. Then, the reaction was quenched with MeOH. The reaction mixture was transferred to a centrifuge tube, shaken and centrifuged fully, and the yield was extracted by HPLC using the supernatant.

General procedure for the catalyst-free and solvent-free Michael addition

Nitroalkene (0.36 mmol), 1,3-dicarbonyl compound (0.30 mmol) and quartz sand (2.25 g) were added in a mortar, mixed thoroughly and ground well (intermittently) at rt, and then even placed overnight until reacted completely. The mixture was purified directly by flash column chromatography to give the product without any pretreatment. For the simpler purification procedure, the reaction mixture was transferred to a sand core funnel in which a thin layer of column-layer chromatographic silica gel was laid down in advance, and then the elution process was performed. First, redundant β-nitrostyrene was directly eluted with dichloromethane, and then the pure product was obtained by using dichloromethane/methanol 5:1 v/v as eluent.

No Michael product was detected in the reaction of β-nitrostyrene and cyclohexane-1,3-dione, and another product was only obtained by a tandem process (Scheme S1, 73% yield).

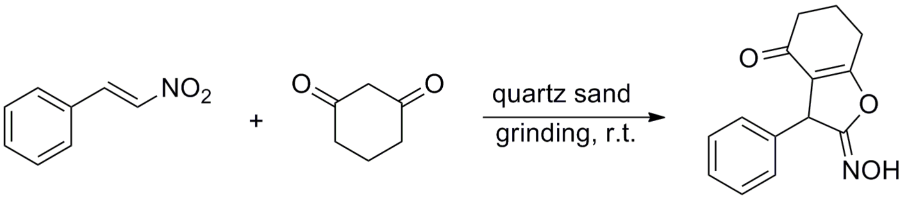

Scheme S1: The tandem reaction of β-nitrostyrene and cyclohexane-1,3-dione

Investigation of the reactivity of various nitroalkenes

The reactivity of various nitroalkenes with 1,3-cyclopentanedione was studied (Table S1). Compared with β-nitrostyrene (Table S1, entry 1), its derivatives showed lower reactivity (Table S1, entries 2–4,6–12) with the exception of 1-chloro-3-(2-nitrovinyl)benzene (Table S1, entry 5), and especially low in the cases of (2-nitroprop-1-en-1-yl)benzene (Table S1, entry 12) and 1-nitro-2-(2-nitrovinyl)benzene (Table S1, entry 9), where steric effects or electronic effects were probably the main reasons.

Table S1: The reactivity of different nitroalkenes with 1,3-cyclopentanedione.[a]

Entry	Acceptor	Conv.[%][b]	Entry	Acceptor	Conv.[%][b]
1	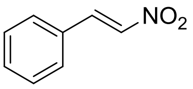	99	8	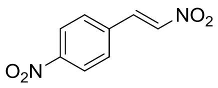	37
2	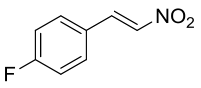	55	9	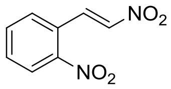	13
3	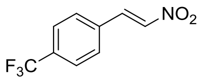	76	10	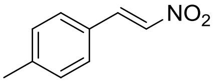	94
4	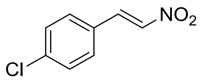	52	11	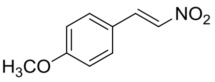	82
5	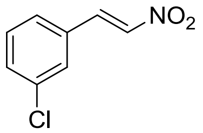	99	12	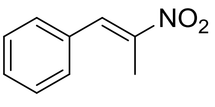	< 1
6	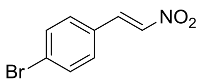	25	13	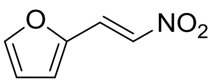	74
7	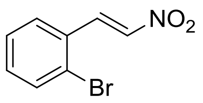	25			

[a]Conditions: acceptor (0.12 mmol), 1,3-cyclopentanedione (0.1 mmol), quartz sand (0.75 g), ground for 10 min and stood for 10 min. [b]Conv. referring to the percentage conversion of 1,3-cyclopentanedione as determined by HPLC.

Analytical data of products

1,3-Cyclopentanedione (*material*)

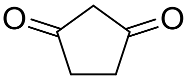

^{1}H NMR (400 MHz, DMSO-d_6) δ 12.07 (s, 1 H), 5.09 (s, 1 H), 2.38 (s, 4 H); ^{13}C NMR (100 MHz, DMSO-d_6) δ 105.46, 31.70.

2-(2-Nitro-1-phenylethyl)cyclopentane-1,3-dione (3a)

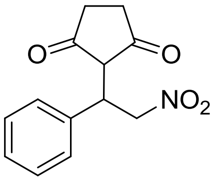

^{1}H NMR (400 MHz, DMSO-d_6) δ 12.31 (s, 1 H), 7.34–7.19 (m, 5 H), 5.23–5.12 (m, 2 H), 4.47 (t, J = 8.2 Hz, 1 H), 2.38 (s, 4 H); ^{13}C NMR (100 MHz, DMSO-d_6) δ 140.19, 128.99, 128.08, 127.42, 114.66, 77.05, 38.53, 30.61; HRMS–ESI (*m/z*): $[M + Na]^+$ calcd for $C_{13}H_{13}NO_4$, 270.0737; found, 270.0738.

2-(1-(4-Fluorophenyl)-2-nitroethyl)cyclopentane-1,3-dione (3b)

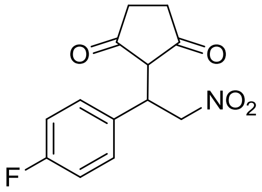

^{1}H NMR (400 MHz, DMSO-d_6) δ 7.38–7.34 (m, 2 H), 7.12 (t, J = 8.8 Hz, 2 H), 5.20–5.09 (m, 2 H), 4.46 (t, J = 8.2 Hz, 1 H), 2.38 (s, 4 H); ^{13}C NMR (100 MHz, DMSO-d_6) δ 162.83, 160.42, 136.34 (d, J = 3.1 Hz), 130.03 (d, J = 8.1 Hz), 115.83, 115.62, 114.45, 77.04, 37.80, 30.59; HRMS–ESI (*m/z*): $[M + H]^+$ calcd for $C_{13}H_{12}FNO_4$: 266.0823, found: 266.0834.

2-(2-Nitro-1-(4-(trifluoromethyl)phenyl)ethyl)cyclopentane-1,3-dione (3c)

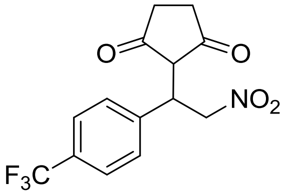

^{1}H NMR (400 MHz, DMSO-d_6) δ 7.67 (d, J = 8.2 Hz, 2 H), 7.56 (d, J = 8.2 Hz, 2 H), 5.24 (d, J = 8.2 Hz, 2 H), 4.57 (t, J = 8.1 Hz, 1 H), 2.39 (s, 4 H); ^{13}C NMR (100 MHz, DMSO-d_6) δ 144.92, 128.92, 128.29, 127.97, 126.02–125.86 (m), 123.31, 113.80, 76.41, 38.24, 30.64; HRMS–ESI (*m/z*): $[M + H]^+$ calcd for $C_{14}H_{12}F_3NO_4$, 316.0791; found, 316.0802.

2-(1-(4-Chlorophenyl)-2-nitroethyl)cyclopentane-1,3-dione (3d)

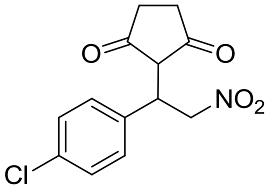

^{1}H NMR (400 MHz, DMSO-d_6) δ 7.35 (s, 4 H), 5.18–5.15 (m, 2 H), 4.46 (t, J = 8.2 Hz, 1 H), 2.39 (s, 4 H); ^{13}C NMR (100 MHz, DMSO-d_6) δ 139.14, 132.11, 129.97, 128.96, 114.19, 76.75, 37.88, 30.63; HRMS–ESI (*m/z*): $[M + Na]^+$ calcd for $C_{13}H_{12}ClNO_4$, 304.0347; found, 304.0356.

2-(1-(3-Chlorophenyl)-2-nitroethyl)cyclopentane-1,3-dione (3e)

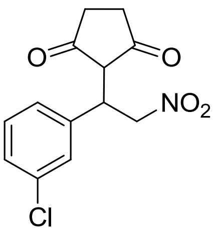

^{1}H NMR (400 MHz, DMSO-d_6) δ 7.39 (s, 1 H), 7.36–7.28 (m, 3 H), 5.20–5.17 (m, 2 H), 4.47 (t, *J* = 8.1 Hz, 1 H), 2.40 (s, 4 H); ^{13}C NMR (100 MHz, DMSO-d_6) δ 142.58, 133.53, 130.92, 127.89, 127.52, 126.89, 113.99, 76.59, 38.12, 30.66; HRMS–ESI (*m*/*z*): $[M + H]^+$ calcd for $C_{13}H_{12}ClNO_4$, 282.0528; found, 282.0533.

2-(1-(4-Bromophenyl)-2-nitroethyl)cyclopentane-1,3-dione (3f)

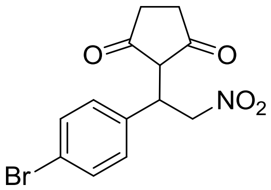

^{1}H NMR (400 MHz, DMSO-d_6) δ 7.49 (d, *J* = 8.4 Hz, 2 H), 7.29 (d, *J* = 8.4 Hz, 2 H), 5.19–5.15 (m, 2 H), 4.45 (t, *J* = 8.2 Hz, 1 H), 2.39 (s, 4 H); ^{13}C NMR (100 MHz, DMSO-d_6) δ 139.07, 131.39, 129.84, 120.11, 113.63, 76.16, 37.43, 30.14; HRMS–ESI (*m*/*z*): $[M + Na]^+$ calcd for $C_{13}H_{12}BrNO_4$, 347.9842; found, 347.9840.

2-(1-(2-Bromophenyl)-2-nitroethyl)cyclopentane-1,3-dione (3g)

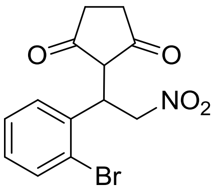

^{1}H NMR (400 MHz, DMSO-d_6) δ 7.60 (d, *J* = 8.0 Hz, 1 H), 7.54 (d, *J* = 7.8 Hz, 1 H), 7.34 (t, *J* = 7.5 Hz, 1 H), 7.18 (t, *J* = 7.6 Hz, 1 H), 5.23–5.16 (m, 1 H), 4.95–4.90 (m, 2 H), 2.41 (s, 4 H); ^{13}C NMR (100 MHz, DMSO-d_6) δ 138.61, 133.30, 130.20, 129.59, 128.47, 124.07, 113.11, 75.82, 38.12, 30.69; HRMS–ESI (*m*/*z*): $[M + H]^+$ calcd for $C_{13}H_{12}BrNO_4$, 326.0022; found, 326.0023.

2-(2-Nitro-1-(4-nitrophenyl)ethyl)cyclopentane-1,3-dione (3h)

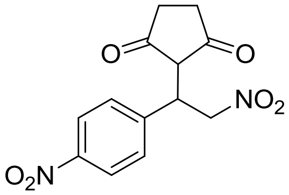

^{1}H NMR (400 MHz, DMSO-d_6) δ 8.17 (d, *J* = 8.7 Hz, 2 H), 7.61 (d, *J* = 8.7 Hz, 2 H), 5.31–5.20 (m, 2 H), 4.61 (t, *J* = 8.1 Hz, 1 H), 2.40 (s, 4 H); ^{13}C NMR (100 MHz, DMSO-d_6) δ 147.81, 146.98, 129.38, 124.19, 113.46, 76.10, 38.15, 30.66; HRMS–ESI (*m*/*z*): $[M + H]^+$ calcd for $C_{13}H_{12}N_2O_6$, 293.0768; found, 293.0774.

2-(2-Nitro-1-(2-nitrophenyl)ethyl)cyclopentane-1,3-dione (3i)

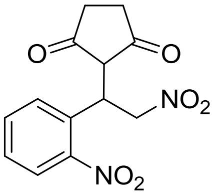

^{1}H NMR (400 MHz, DMSO-d_6) δ 7.86 (d, J = 8.1 Hz, 1 H), 7.75 (d, J = 7.7 Hz, 1 H), 7.67 (t, J = 7.5 Hz, 1 H), 7.49 (t, J = 7.6 Hz, 1 H), 5.38–5.33 (m, 1 H), 5.13–5.08 (m, 1 H), 4.97–4.93 (m, 1 H), 2.37 (s, 4 H); ^{13}C NMR (100 MHz, DMSO-d_6) δ 149.03, 133.21, 133.14, 130.09, 128.49, 124.16, 112.76, 75.24, 32.98, 30.10; HRMS–ESI (m/z): $[M + H]^+$ calcd for $C_{13}H_{12}N_2O_6$, 293.0768; found, 293.0779.

2-(2-Nitro-1-(p-tolyl)ethyl)cyclopentane-1,3-dione (3j)

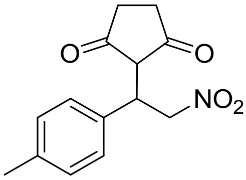

^{1}H NMR (400 MHz, DMSO-d_6) δ 6.97 (d, J = 7.9 Hz, 2 H), 6.85 (d, J = 7.9 Hz, 2 H), 4.95–4.85 (m, 2 H), 4.18 (t, J = 8.2 Hz, 1 H), 2.14 (s, 4 H), 2.00 (s, 3 H); ^{13}C NMR (100 MHz, DMSO-d_6) δ 137.16, 136.56, 129.52, 127.94, 114.80, 77.15, 38.18, 30.59, 21.02; HRMS–ESI (m/z): $[M + H]^+$ calcd for $C_{14}H_{15}NO_4$, 262.1074; found, 262.1085.

2-(1-(4-Methoxyphenyl)-2-nitroethyl)cyclopentane-1,3-dione (3k)

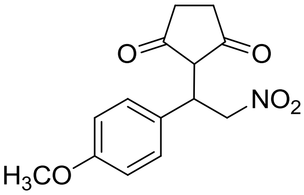

^{1}H NMR (400 MHz, DMSO-d_6) δ 7.25 (d, J = 8.5 Hz, 2 H), 6.85 (d, J = 8.5 Hz, 2 H), 5.17–5.06 (m, 2 H), 4.41 (t, J = 8.2 Hz, 1 H), 3.71 (s, 3 H), 2.38 (s, 4 H); ^{13}C NMR (100 MHz, DMSO-d_6) δ 158.67, 132.13, 129.17, 114.94, 114.35, 77.35, 55.47, 37.84, 30.60; HRMS–ESI (m/z): $[M + H]^+$ calcd for $C_{13}H_{15}NO_5$, 278.1023; found, 278.1032.

2-(2-Nitro-1-phenylpropyl)cyclopentane-1,3-dione (3l)

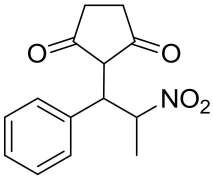

^{1}H NMR (400 MHz, DMSO-d_6) δ 7.43–7.18 (m, 5 H), 5.81–5.70 (m, 1 H), 4.11–4.04 (m, 1 H), 2.39 (s, 0.71 H), 2.32 (s, 3.17), 1.45 (d, J = 6.5 Hz, 0.56 H), 1.26 (d, J = 6.7 Hz, 2.49 H); ^{13}C NMR (100 MHz, DMSO-d_6) δ 140.61, 139.92, 129.14, 128.95, 128.87, 128.13, 127.57, 127.50, 115.35, 114.66, 85.34, 83.52, 45.59, 45.42, 30.48, 19.25, 19.05; HRMS–ESI (m/z): $[M + H]^+$ calcd for $C_{14}H_{15}NO_4$, 262.1074; found, 262.1081.

2-(1-(Furan-2-yl)-2-nitroethyl)cyclopentane-1,3-dione (3m)

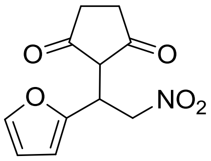

^{1}H NMR (400 MHz, DMSO-d_6) δ 7.52 (s, 1 H), 6.34 (t, J = 2 Hz, 1 H), 6.15 (d, J = 3.1 Hz, 1 H), 5.14–5.02 (m, 2 H), 4.61 (t, J = 7.9 Hz, 1 H), 2.41 (s, 4 H); ^{13}C NMR (100 MHz, DMSO-d_6) δ 151.81, 142.07, 111.52, 110.54, 106.34, 74.92, 31.74, 30.22; HRMS–ESI (m/z): $[M + H]^+$ calcd for $C_{11}H_{11}NO_5$, 238.0710; found, 238.0714.

3-(2-Nitro-1-phenylethyl)furan-2,4(3H,5H)-dione (3n)

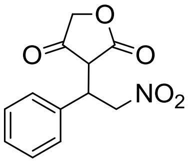

^{1}H NMR (400 MHz, DMSO-d_6) δ 7.38–7.24 (m, 5 H), 5.23 (dd, J = 13.3, 8.9 Hz, 1 H), 5.14 (dd, J = 13.3, 7.5 Hz, 1 H), 4.64 (s, 2 H), 4.51 (t, J = 8.2 Hz, 1 H); ^{13}C NMR (100 MHz, DMSO-d_6) δ 176.43, 174.33, 139.41, 129.14, 128.02, 127.75, 97.56, 76.84, 67.16, 38.13; HRMS–ESI (m/z): $[M + Na]^+$ calcd for $C_{12}H_{11}NO_5$, 272.0529; found, 272.0531.

2-(2-Nitro-1-phenylethyl)-1-phenylbutane-1,3-dione (3o)

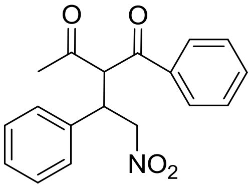

^{1}H NMR (400 MHz, $CDCl_3$) δ 8.03–7.14 (m, 10 H), 5.18 (t, J = 9.8 Hz, 1 H), 4.88–4.64 (m, 2 H), 4.56–4.40 (m, 1 H), 2.23 (s, 1.33 H), 1.94 (s, 1.48 H); ^{13}C NMR (100 MHz, $CDCl_3$) δ 201.67, 200.77, 194.06, 193.75, 136.53, 136.30, 136.17, 136.00, 134.49, 134.10, 129.28, 129.10, 129.03, 128.98, 128.56, 128.20, 128.15, 128.03, 78.18, 78.08, 65.36, 64.77, 43.44, 43.30, 29.78, 28.53; HRMS–ESI (m/z): $[M + Na]^+$ calcd for $C_{18}H_{17}NO_4$, 334.1050; found, 334.1064.

Ethyl 2-benzoyl-4-nitro-3-phenylbutanoate (3p)

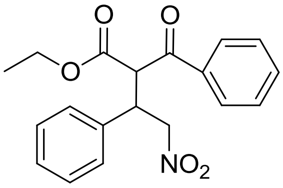

^{1}H NMR (400 MHz, $CDCl_3$) δ 8.06–7.16 (m, 10 H), 4.98–4.90 (m, 2 H), 4.82–4.73 (m, 1 H), 4.52–4.41 (m, 1 H), 4.17 (q, J = 7.2, 7.2 Hz, 1 H), 3.89–3.83 (m, 1 H), 1.17 (t, J = 7.1 Hz, 1.41 H), 0.89 (t, J = 7.1 Hz, 1.74 H); ^{13}C NMR (100 MHz, $CDCl_3$) δ 192.78, 192.74, 167.73, 166.99, 136.77, 136.27, 136.04, 135.85, 134.25, 133.84, 128.96, 128.93, 128.91, 128.75, 128.57, 128.36, 128.30, 128.13, 127.99, 78.00, 62.24, 61.96, 57.04, 56.38, 43.13, 43.11, 13.89, 13.59; HRMS–ESI (m/z): $[M + H]^+$ calcd for $C_{19}H_{19}NO_5$, 342.1336; found, 342.1339.

Ethyl 2-(4-methoxybenzoyl)-4-nitro-3-phenylbutanoate (3q)

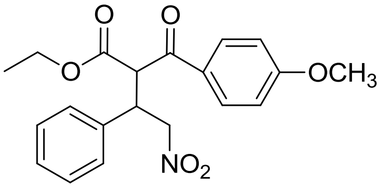

^{1}H NMR (400 MHz, $CDCl_3$) δ 8.05 (d, *J* = 8.9 Hz, 1.11 H), 7.87 (d, *J* = 8.9 Hz, 0.83 H), 7.31–7.19 (m, 5H), 6.92 (dd, *J* = 26.4, 8.9 Hz, 2 H), 4.96–4.87 (m, 2 H), 4.82–4.70 (m, 1 H), 4.50–4.40 (m, 1 H), 4.17 (q, *J* = 6.0, 7.2 Hz, 1 H), 3.90–3.84 (m, 4 H), 1.18 (t, *J* = 7.1 Hz, 1.26 H), 0.90 (t, *J* = 7.1 Hz, 1.82 H); ^{13}C NMR (100 MHz, $CDCl_3$) δ 190.90, 190.84, 167.95, 167.21, 164.48, 164.15, 136.97, 136.41, 131.44, 131.11, 128.92, 128.89, 128.30, 128.05, 127.94, 114.10, 113.97, 78.11, 78.08, 62.14, 61.86, 56.68, 55.99, 55.61, 55.55, 43.14, 13.94, 13.63; HRMS–ESI (*m*/*z*): $[M + H]^+$ calcd for $C_{20}H_{21}NO_6$, 372.1442; found, 372.1448.

2-(2-Nitro-1-phenylethyl)-1,3-diphenylpropane-1,3-dione (3r)

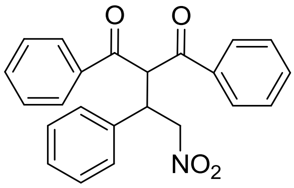

^{1}H NMR (400 MHz, $CDCl_3$) δ 7.86 (dd, *J* = 8.4, 1.1 Hz, 2 H), 7.78 (dd, *J* = 8.3, 1.1 Hz, 2 H), 7.57–7.14 (m, 11 H), 5.84 (d, *J* = 8.0 Hz, 1 H), 5.00 (d, *J* = 6.9 Hz, 2 H), 4.63 (q, *J* = 7.2, 7.6 Hz, 1 H); ^{13}C NMR (100 MHz, $CDCl_3$) δ 194.24, 193.62, 136.80, 136.22, 135.85, 134.06, 133.78, 128.97, 128.95, 128.83, 128.77, 128.60, 128.26, 128.15, 77.31, 59.89, 44.05; HRMS–ESI (*m*/*z*): $[M + H]^+$ calcd for $C_{23}H_{19}NO_4$, 374.1387; found, 374.1390.

3-(1-(Furan-2-yl)-2-nitroethyl)furan-2,4(3H,5H)-dione (3s)

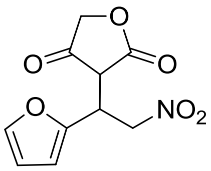

^{1}H NMR (400 MHz, DMSO-d_6) δ 7.56 (d, *J* = 1.3 Hz, 1 H), 6.38 (dd, *J* = 3.0, 2.0 Hz, 1 H), 6.23 (d, *J* = 3.3 Hz, 1 H), 5.14–5.06 (m, 2 H), 4.66–4.62 (m, 3 H); ^{13}C NMR (100 MHz, DMSO-d_6) δ 177.19, 173.81, 151.42, 142.85, 111.05, 107.10, 94.90, 75.07, 67.25, 31.98; HRMS–ESI (*m*/*z*): $[M + Na]^+$ calcd for $C_{10}H_9NO_6$, 262.0322; found, 262.0332.

Spectra

1,3-Cyclopentanedione (*material*)

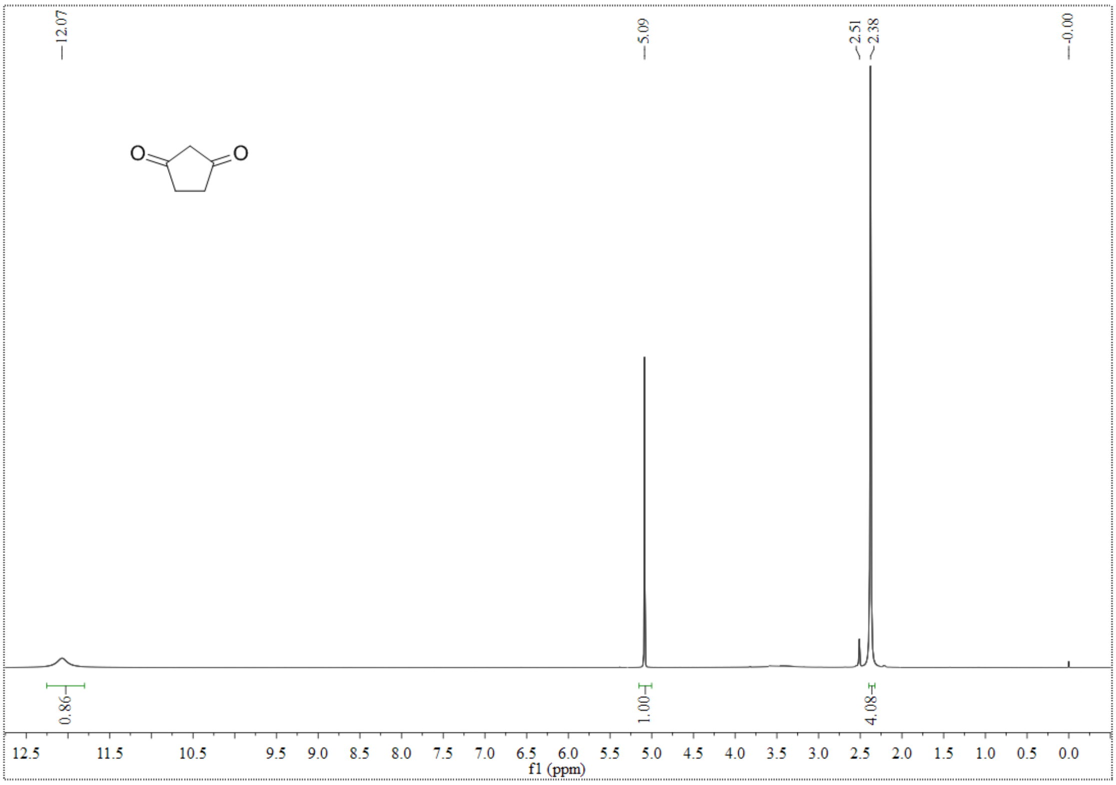

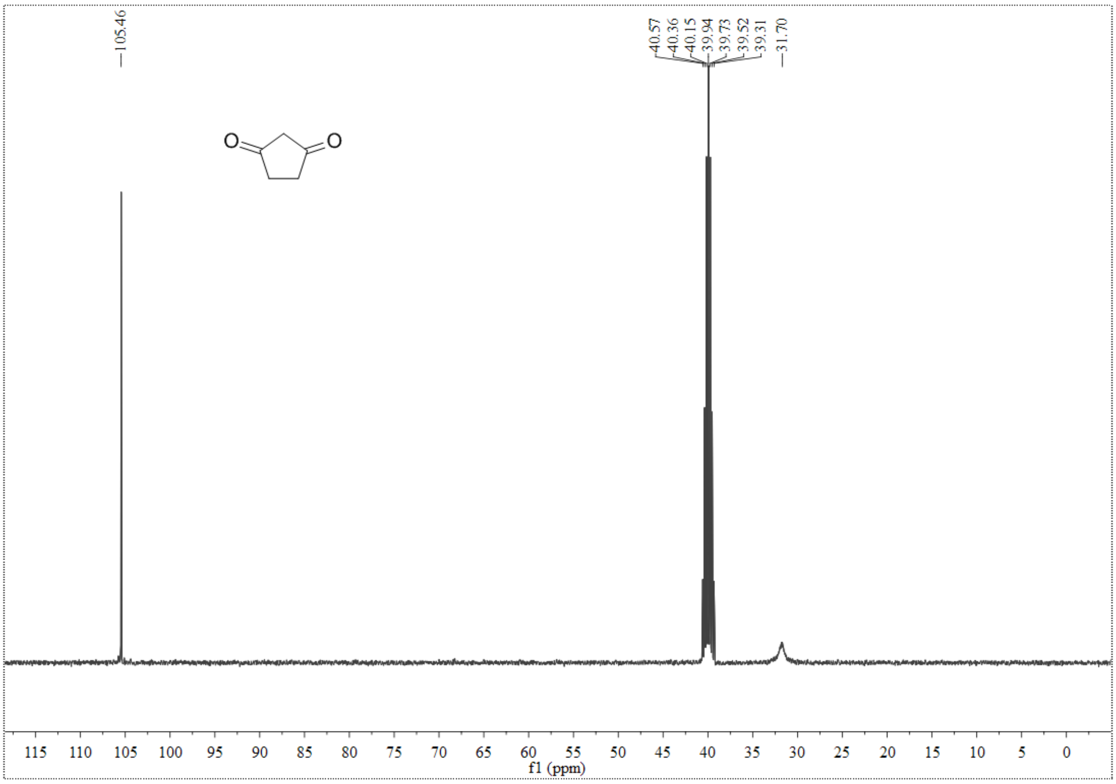

2-(2-Nitro-1-phenylethyl)cyclopentane-1,3-dione (3a)

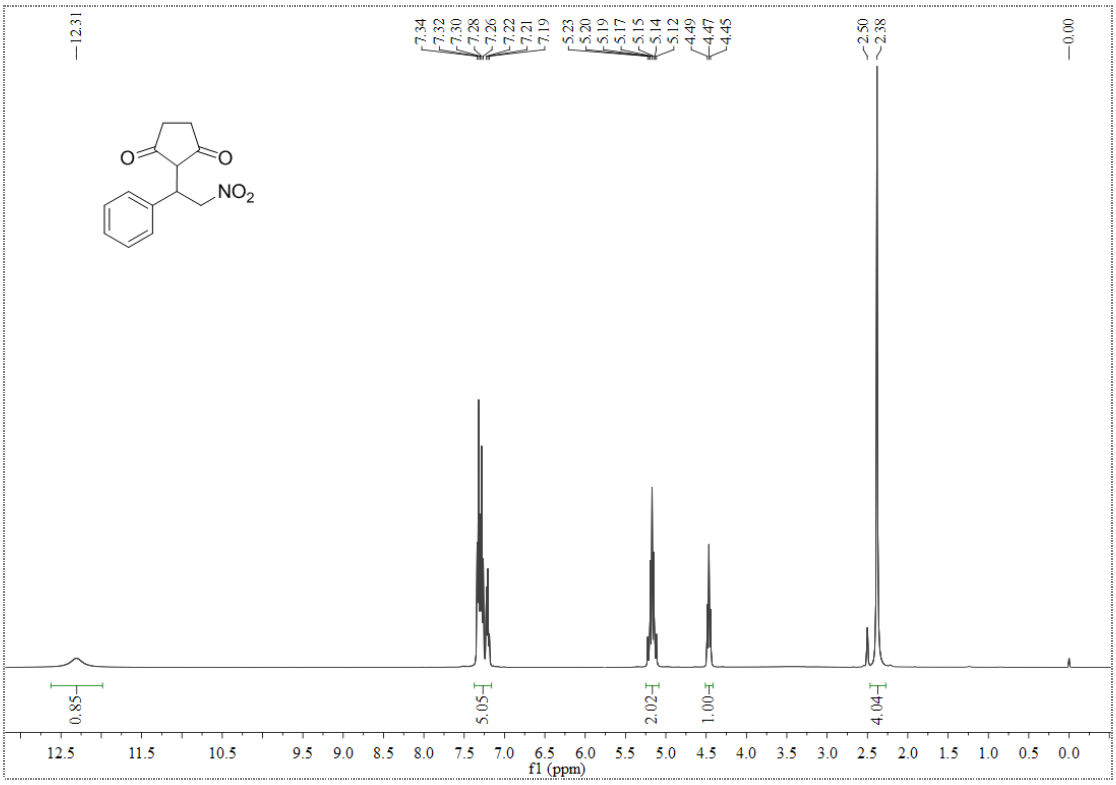

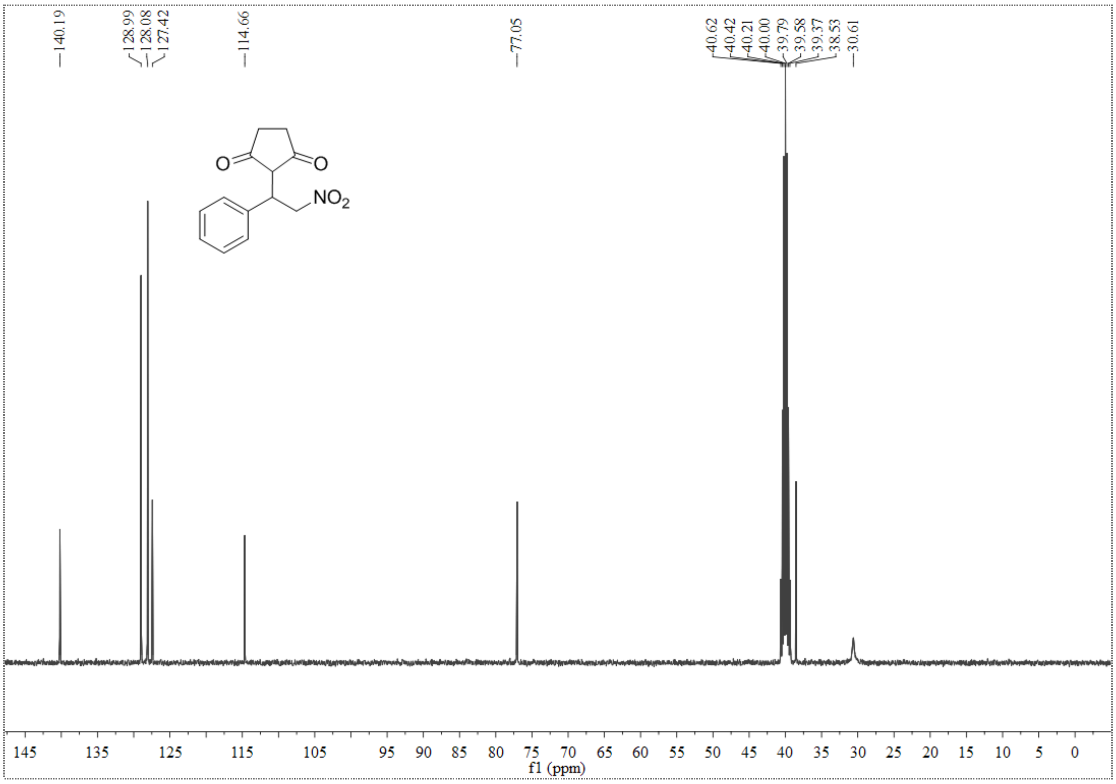

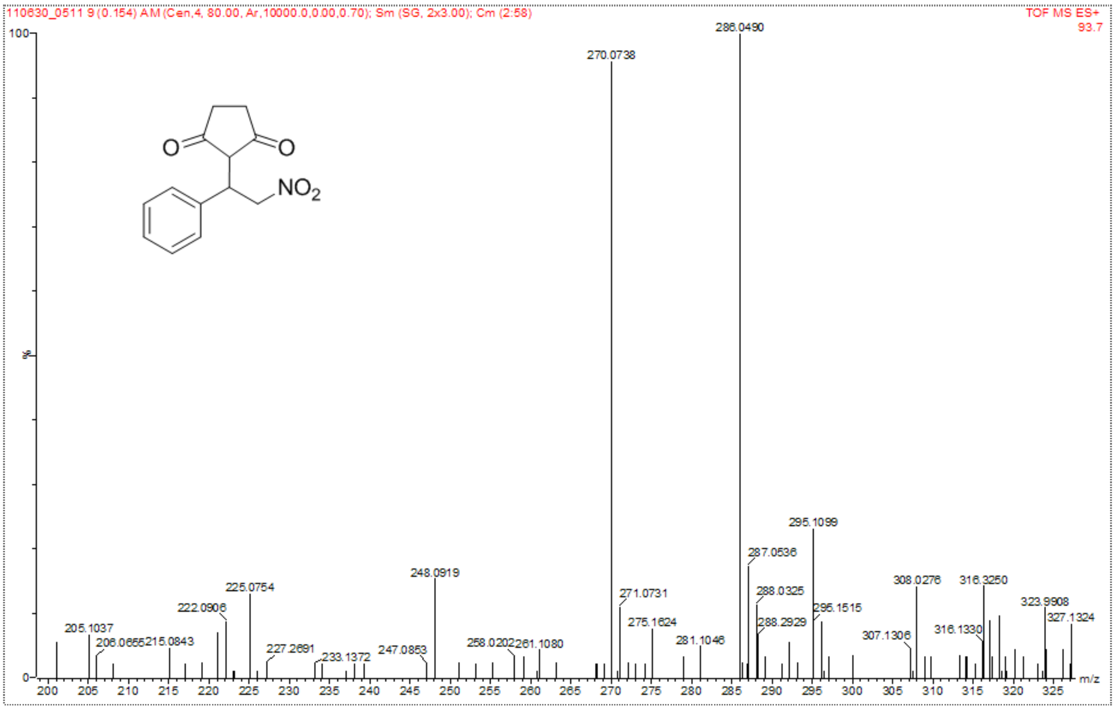

2-(1-(4-Fluorophenyl)-2-nitroethyl)cyclopentane-1,3-dione (3b)

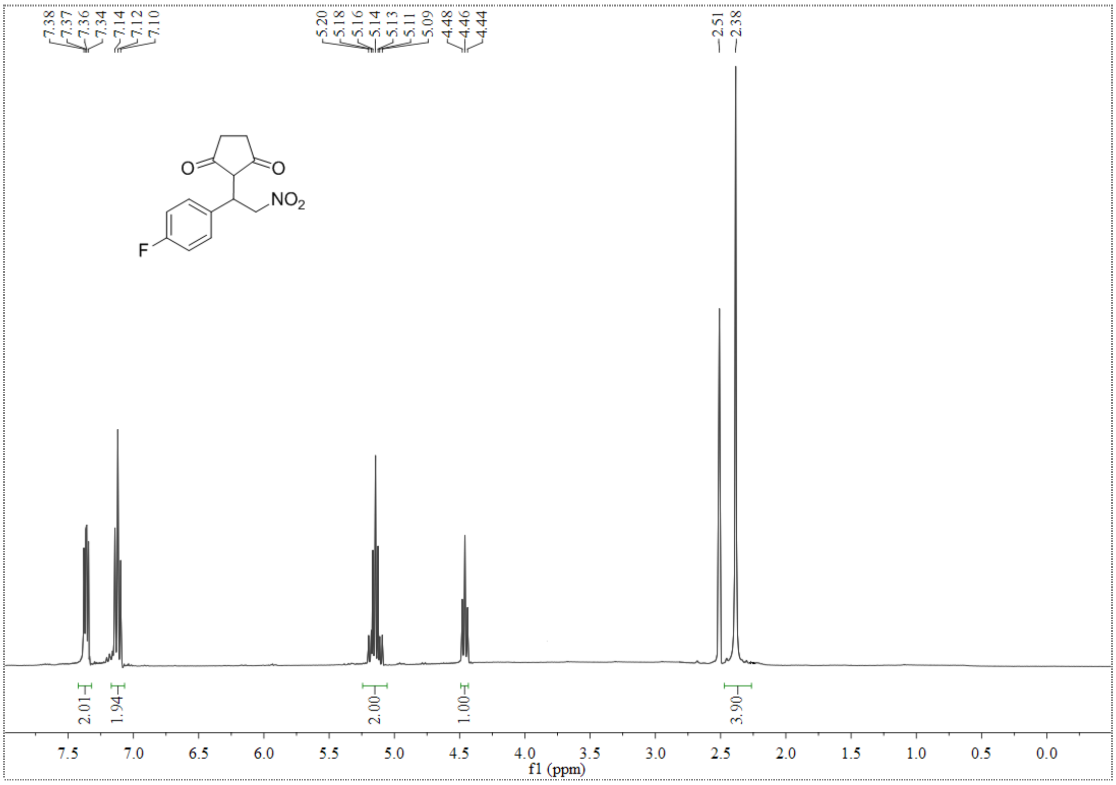

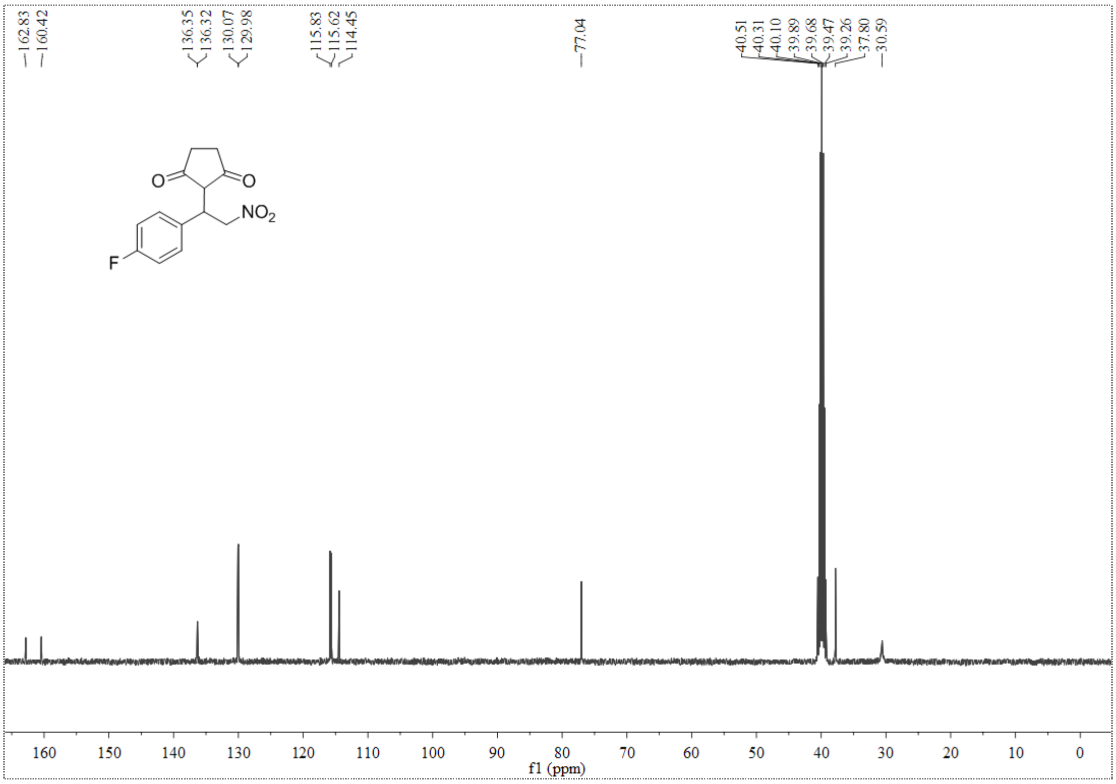
162.83
160.42
136.35
136.32
130.07
129.98
115.83
115.62
114.45
77.04
40.51
40.31
40.10
39.89
39.68
39.47
39.26
37.80
30.59
O
O
NO2
F
f1 (ppm)

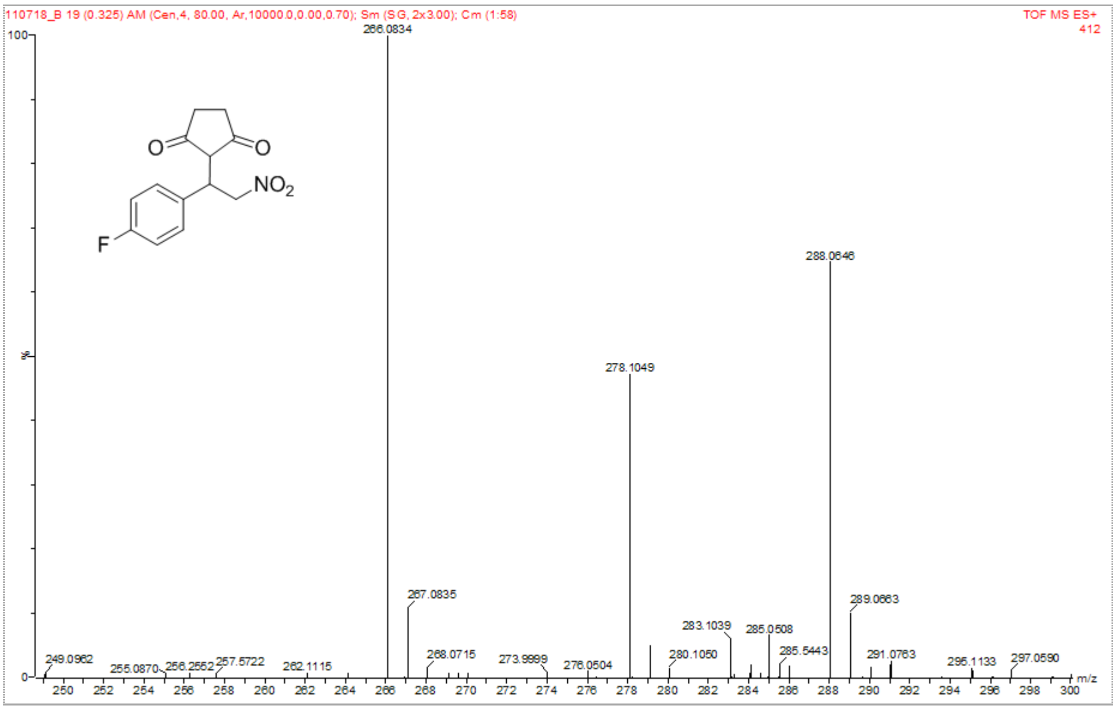
110718_B 19 (0.325) AM (Cen,4, 80.00, Ar,10000.0,0.00,0.70); Sm (SG, 2x3.00); Cm (1:58)
TOF MS ES+
412
266.0834
288.0646
278.1049
267.0835
289.0663
283.1039
285.0508
285.5443
280.1050
291.0763
268.0715
273.9999
276.0504
249.0962
255.0870
256.2552
257.5722
262.1115
295.1133
297.0590
%
m/z
O
O
NO2
F

2-(2-Nitro-1-(4-(trifluoromethyl)phenyl)ethyl)cyclopentane-1,3-dione (3c)

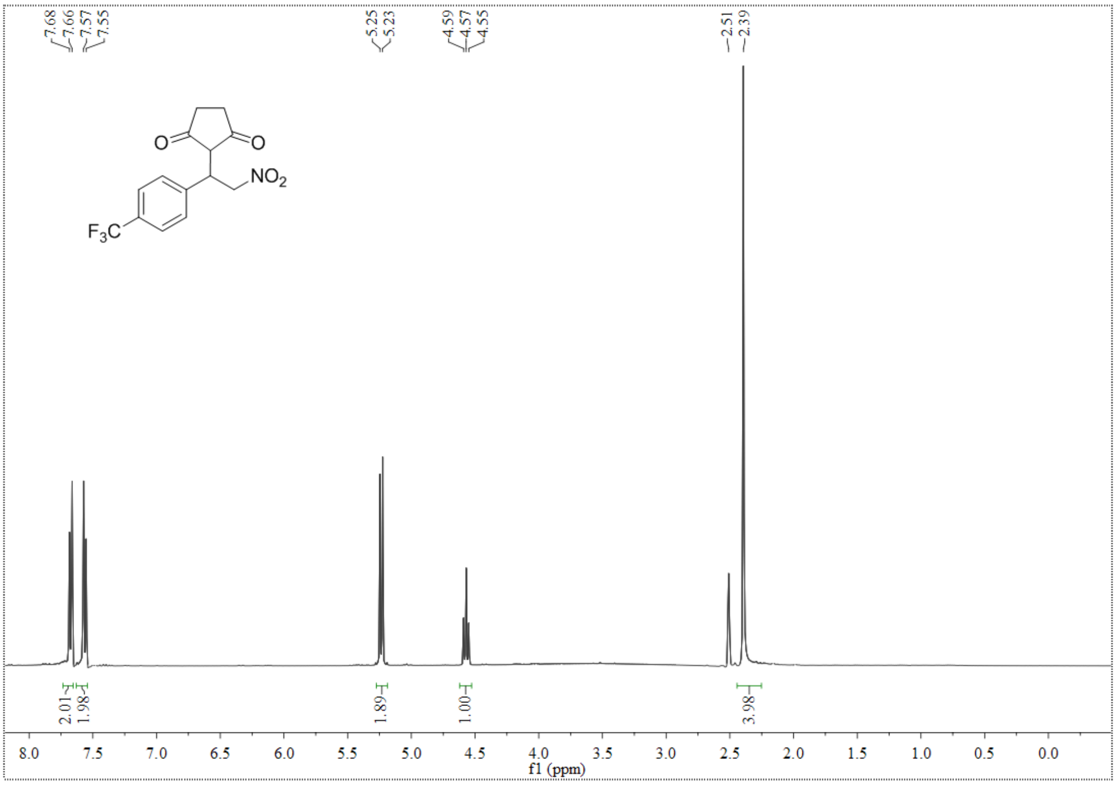

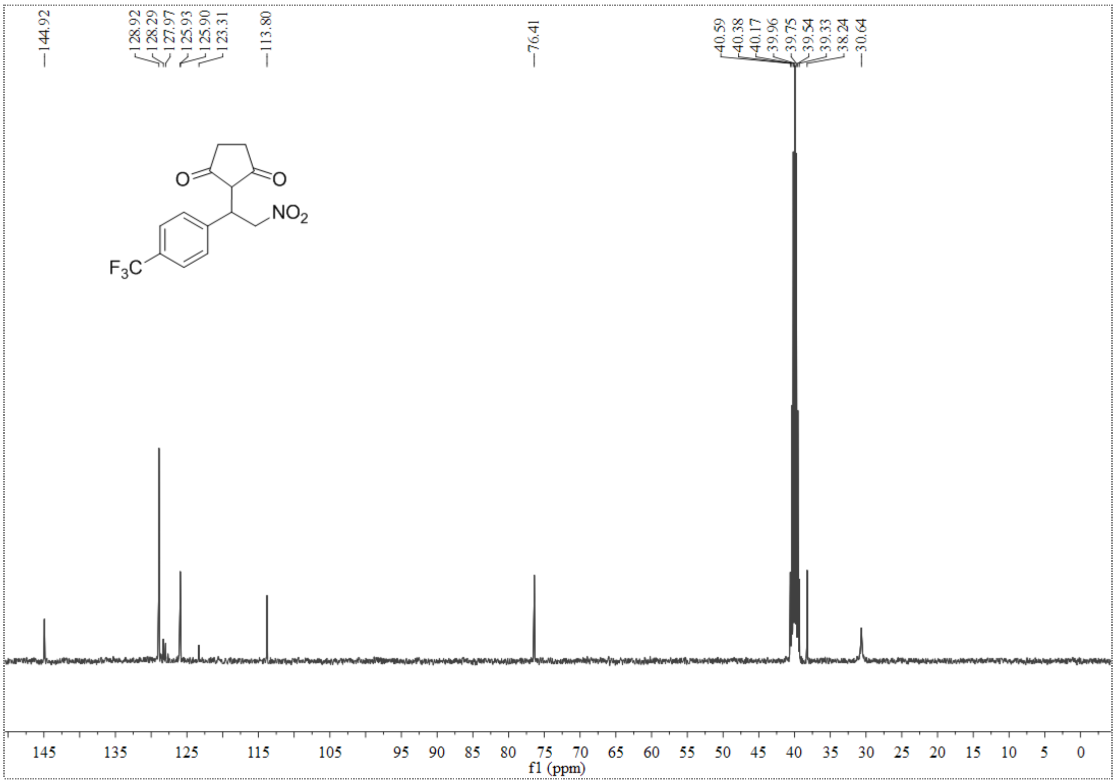

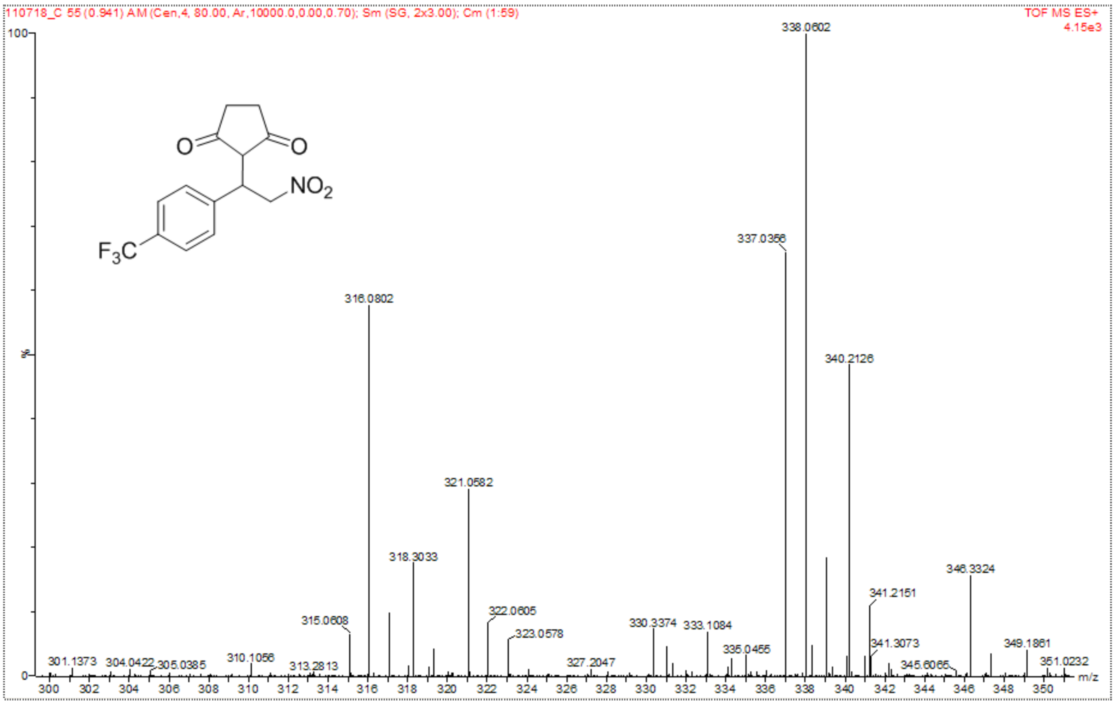

2-(1-(4-Chlorophenyl)-2-nitroethyl)cyclopentane-1,3-dione (3d)

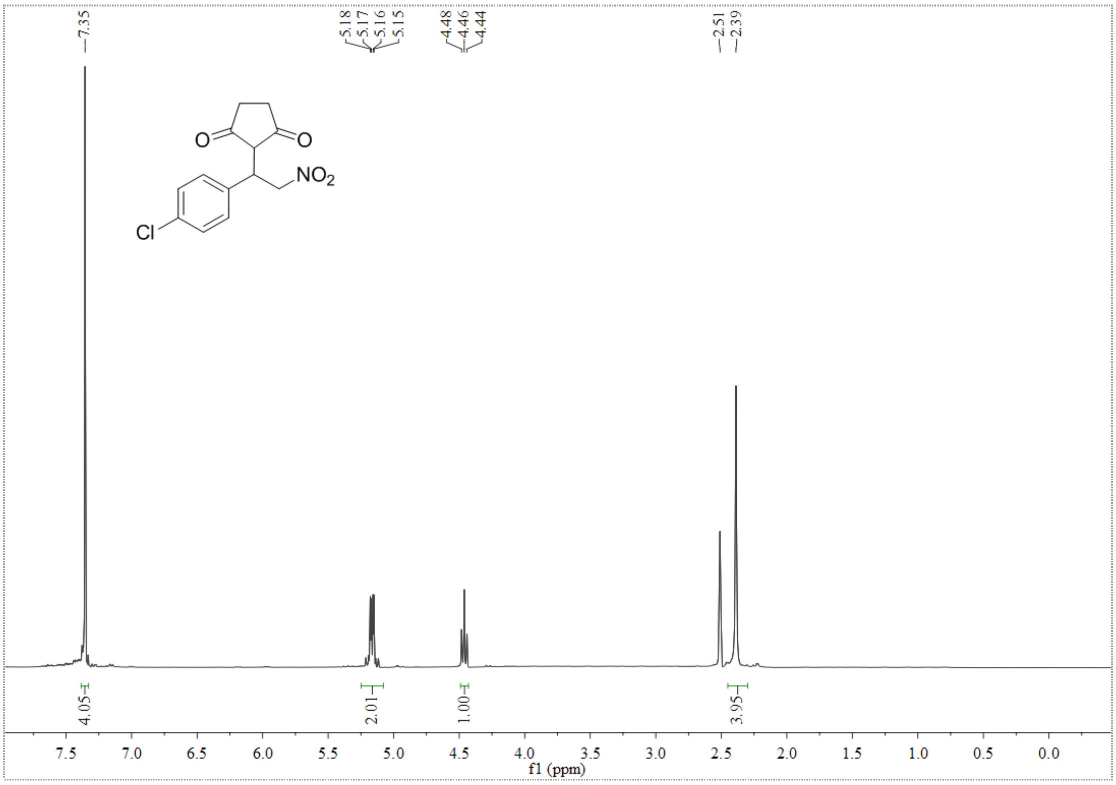

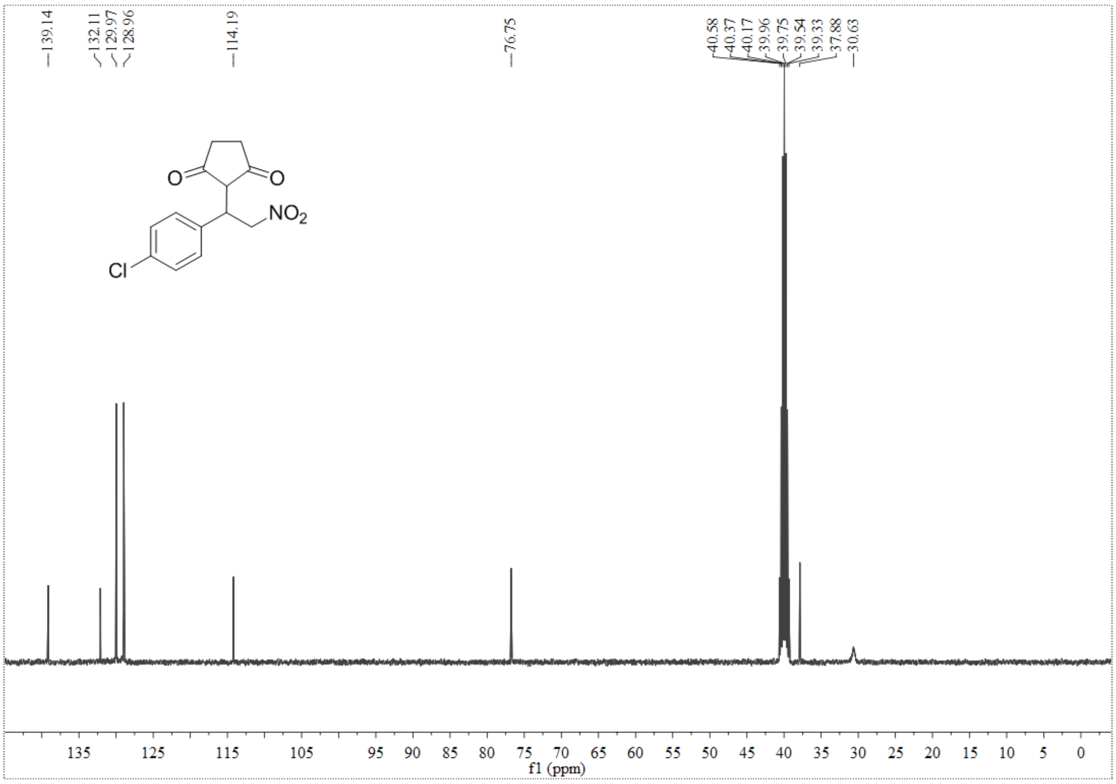
139.14
132.11
129.97
128.96
114.19
76.75
40.58
40.37
40.17
39.96
39.75
39.54
39.33
37.88
30.63
NO2
Cl
O
f1 (ppm)

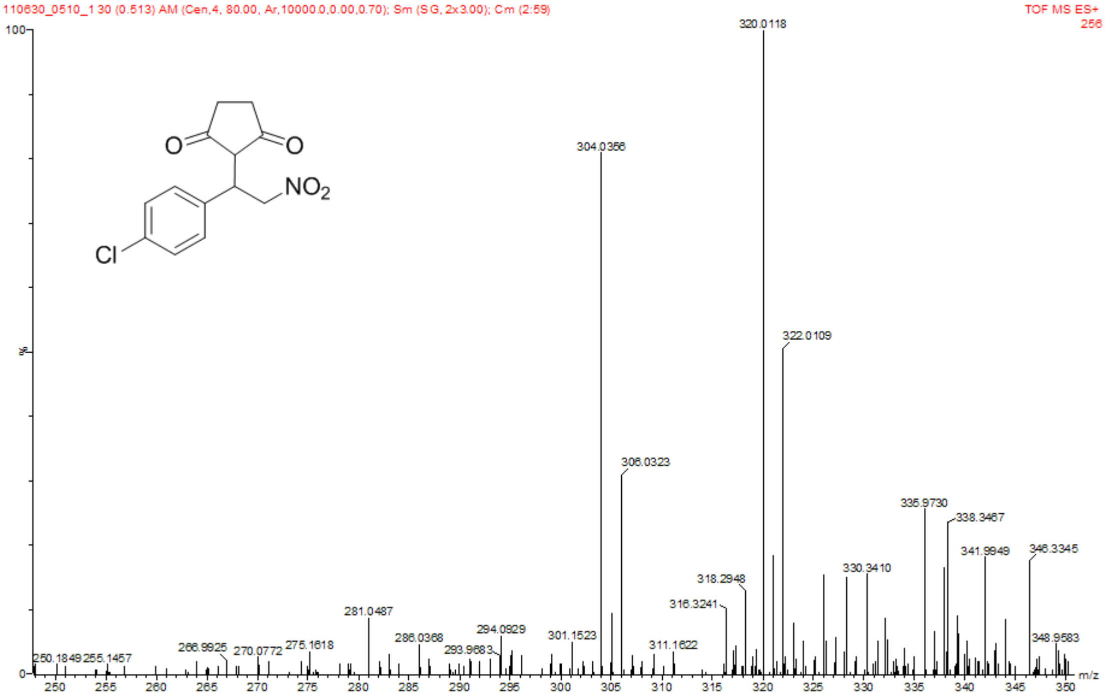
110630_0510_1 30 (0.513) AM (Cen,4, 80.00, Ar,10000.0,0.00,0.70); Sm (SG, 2x3.00); Cm (2:59)
TOF MS ES+
256
320.0118
304.0356
322.0109
306.0323
335.9730
338.3467
341.9949
346.3345
330.3410
318.2948
316.3241
281.0487
348.9583
294.0929
301.1523
311.1622
286.0368
293.9683
266.9925
270.0772
275.1618
250.1849
255.1457
m/z
NO2
Cl

2-(1-(3-Chlorophenyl)-2-nitroethyl)cyclopentane-1,3-dione (3e)

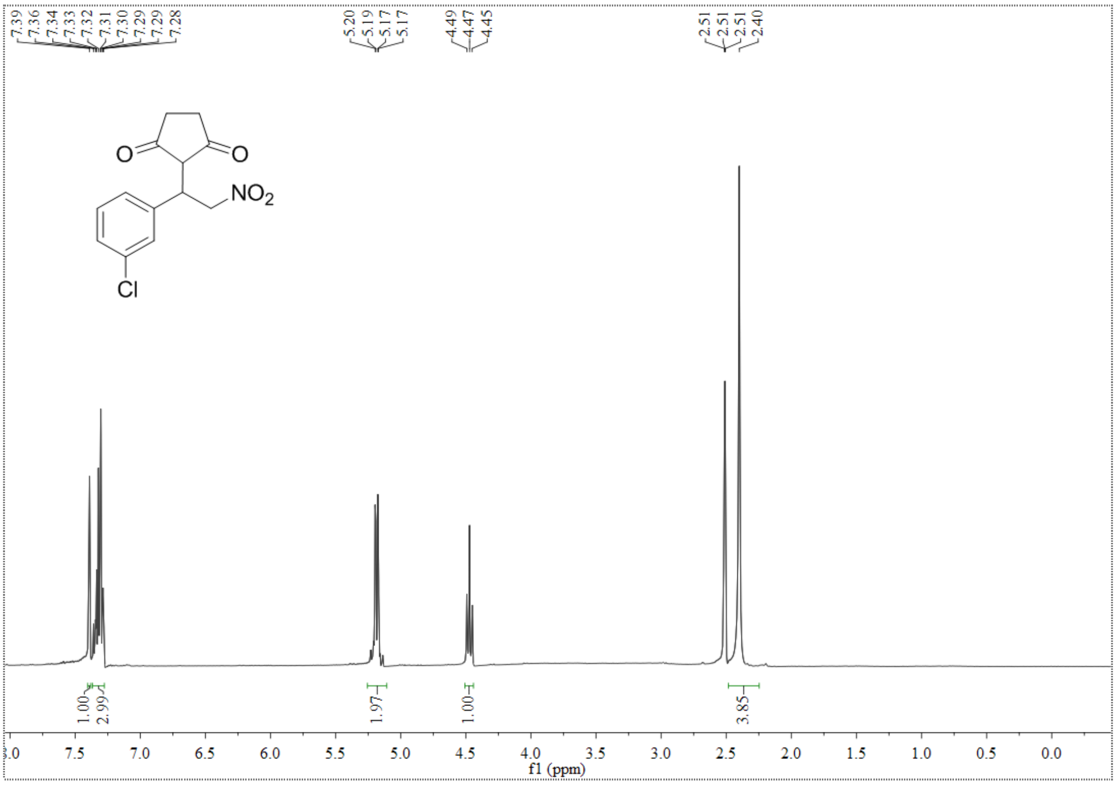

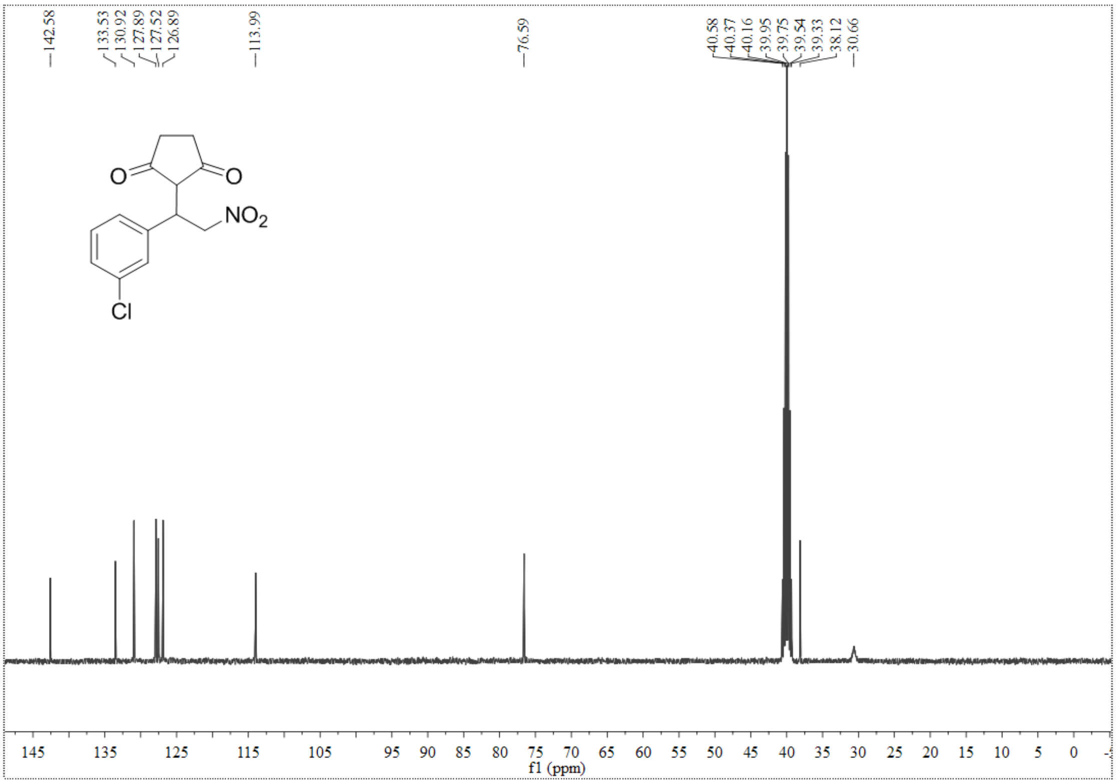

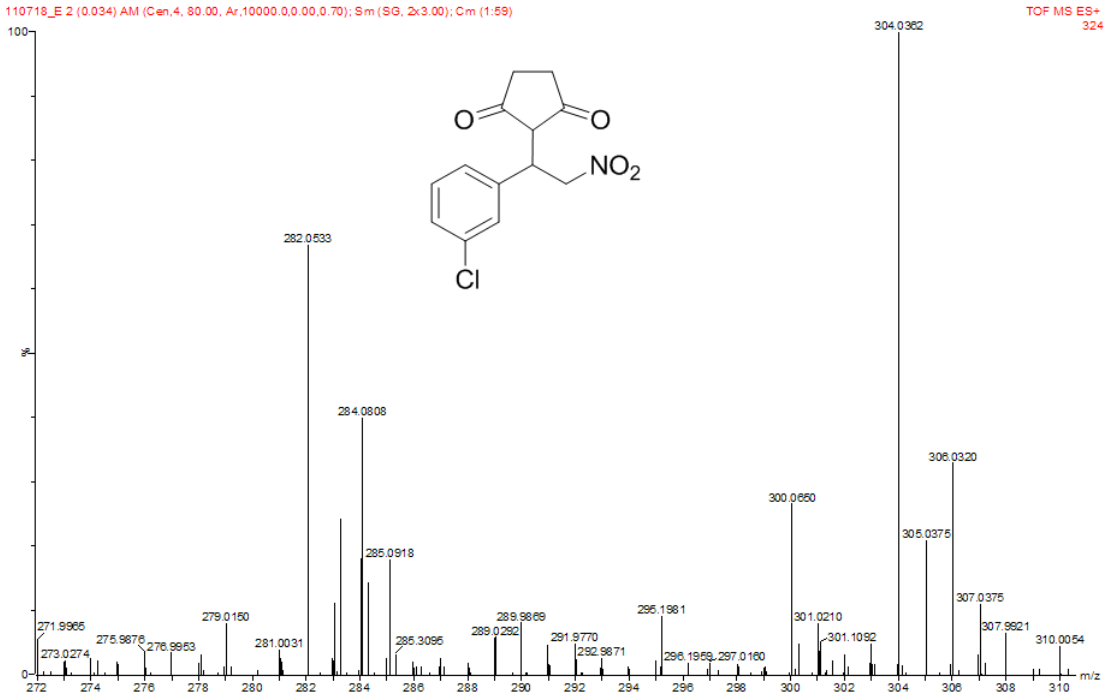

2-(1-(4-Bromophenyl)-2-nitroethyl)cyclopentane-1,3-dione (3f)

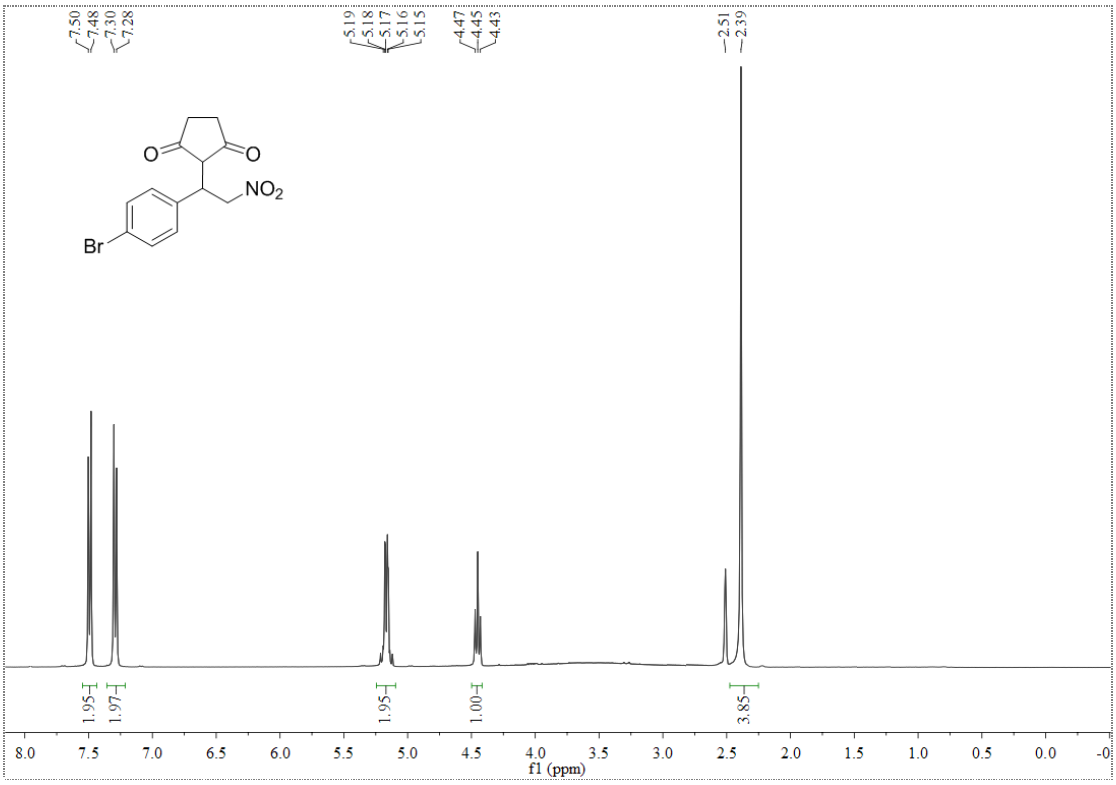

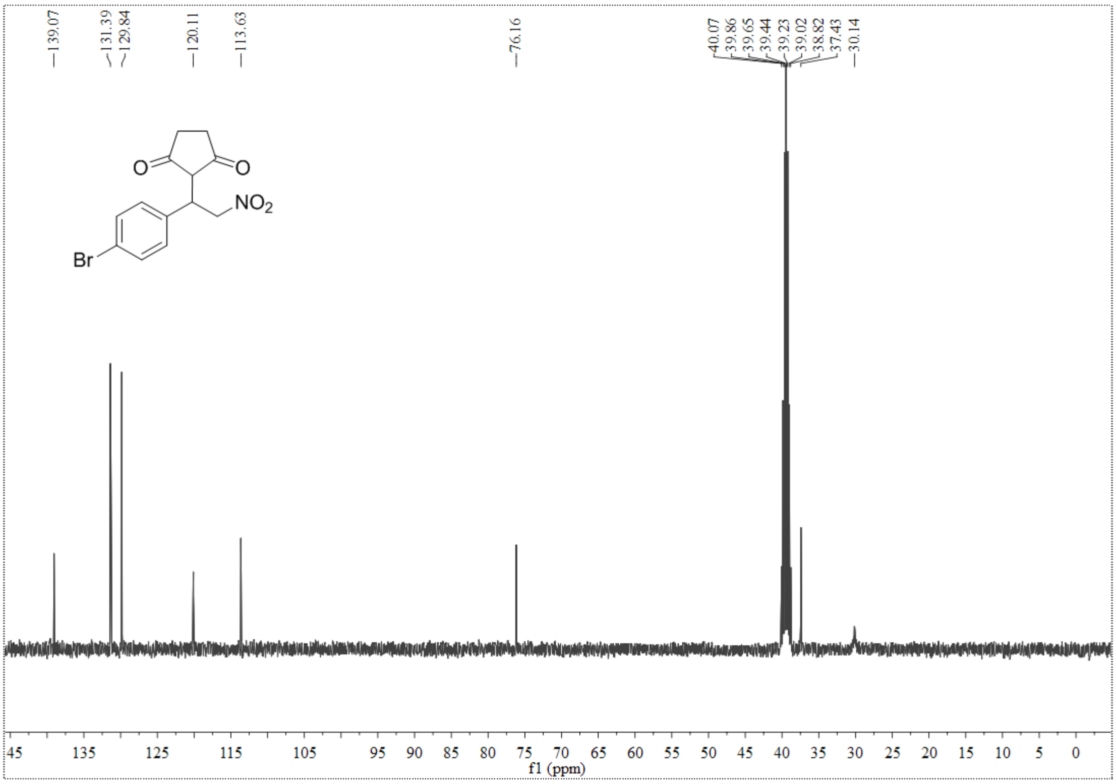
139.07
131.39
129.84
120.11
113.63
76.16
40.07
39.86
39.65
39.44
39.23
39.02
38.82
37.43
30.14
O
O
NO2
Br
f1 (ppm)

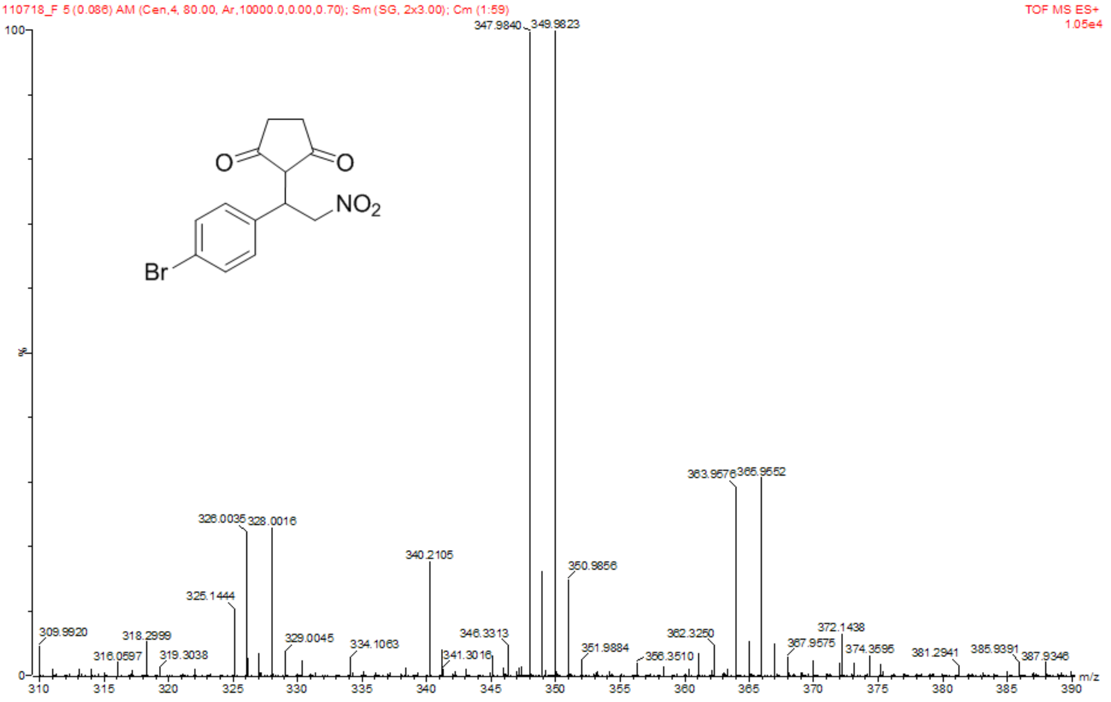
110718_F 5 (0.086) AM (Cen,4, 80.00, Ar,10000.0,0.00,0.70); Sm (SG, 2x3.00); Cm (1:59)
TOF MS ES+
1.05e4
347.9840
349.9823
363.9576
365.9552
326.0035
328.0016
340.2105
350.9856
325.1444
309.9920
318.2999
329.0045
334.1063
346.3313
362.3250
372.1438
367.9575
374.3595
381.2941
385.9391
387.9346
316.0597
319.3038
341.3016
351.9884
356.3510
O
O
NO2
Br
m/z

2-(1-(2-Bromophenyl)-2-nitroethyl)cyclopentane-1,3-dione (3g)

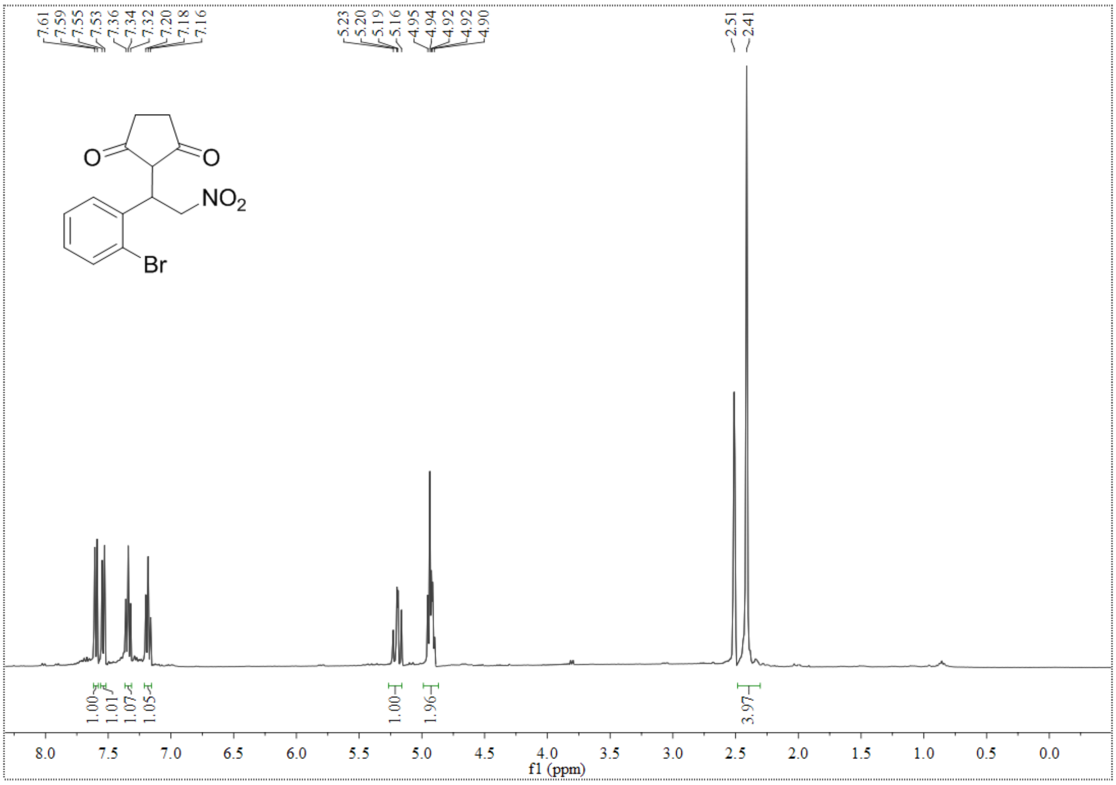

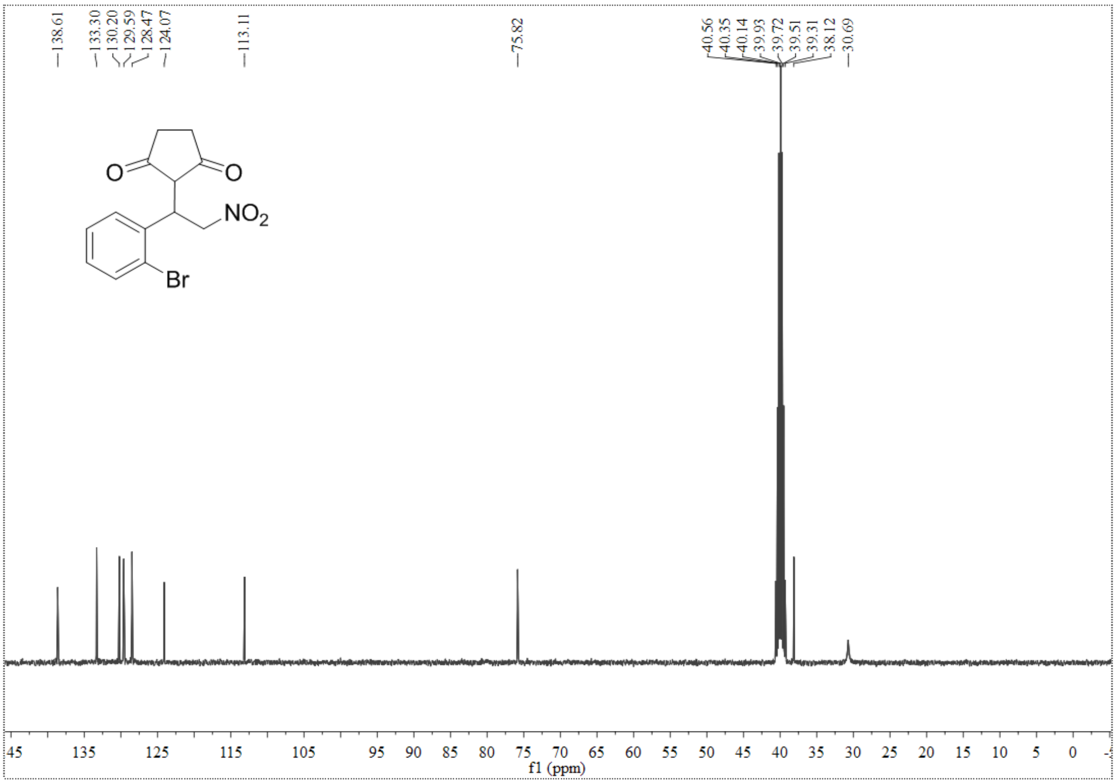

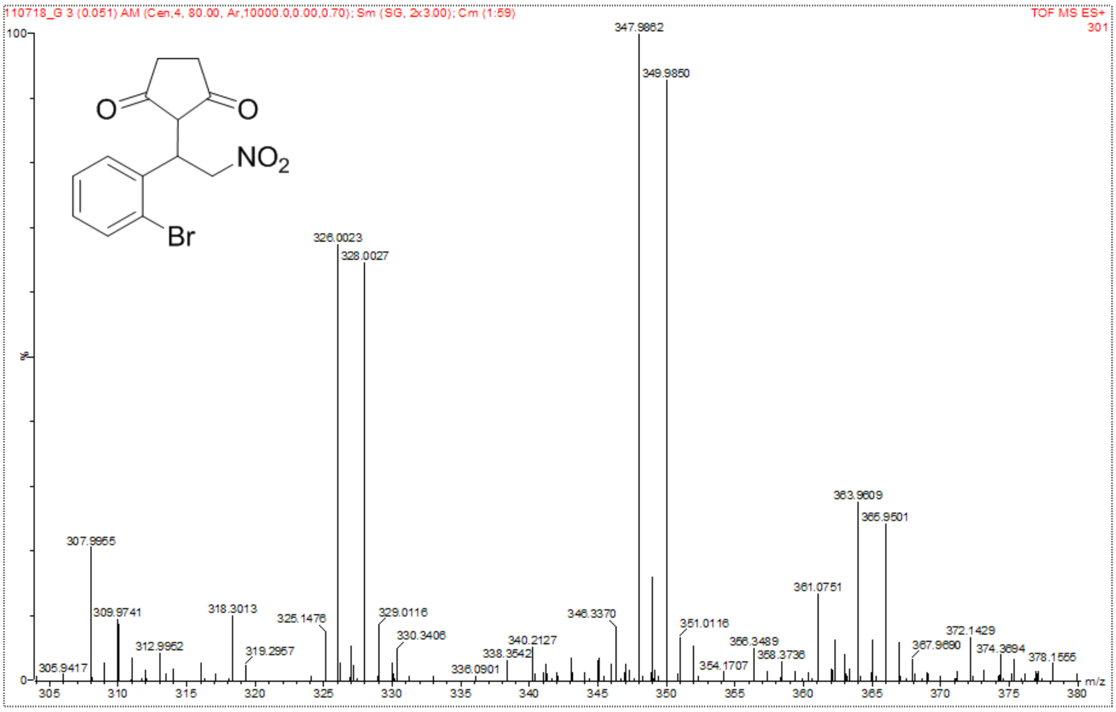

2-(2-Nitro-1-(4-nitrophenyl)ethyl)cyclopentane-1,3-dione (3h)

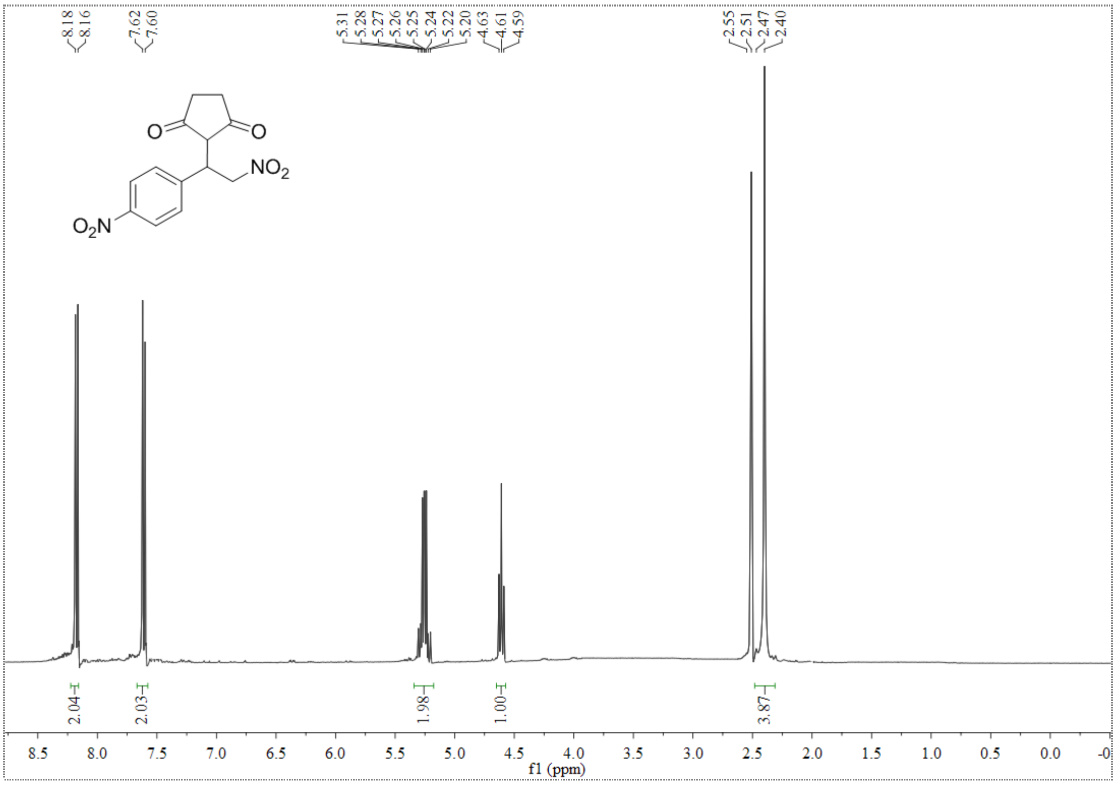

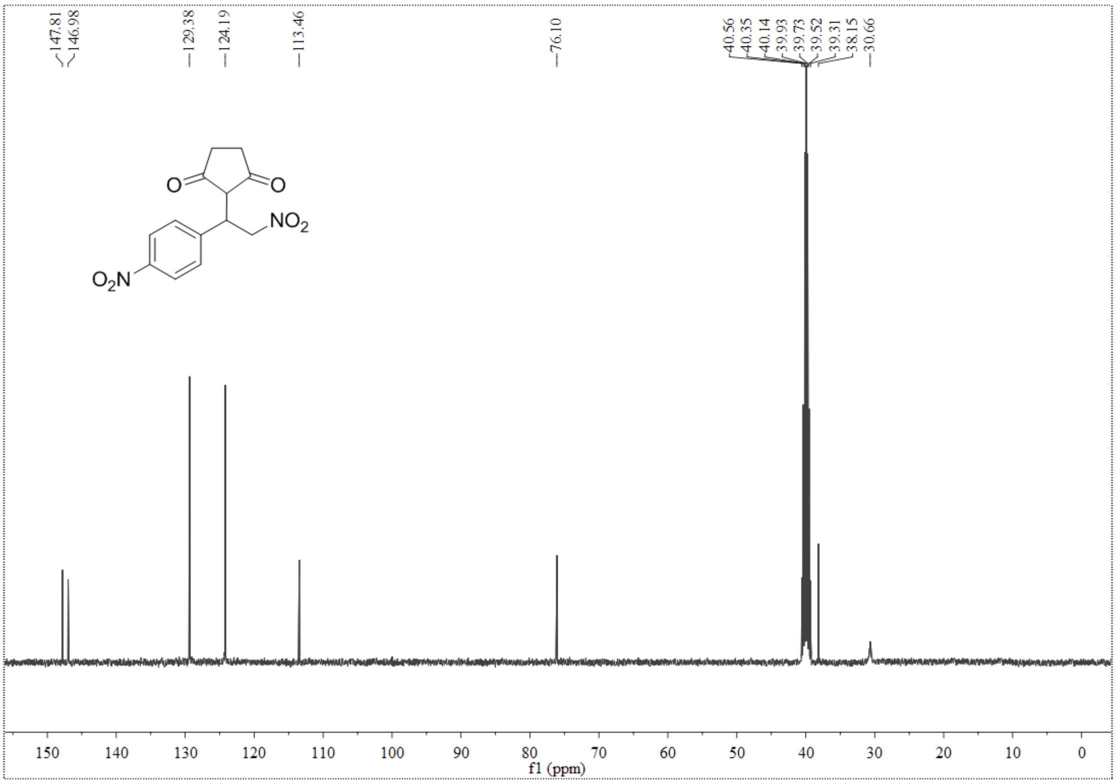
147.81
146.98
129.38
124.19
113.46
76.10
40.56
40.35
40.14
39.93
39.73
39.52
39.31
38.15
30.66
O
O
NO2
O2N
f1 (ppm)

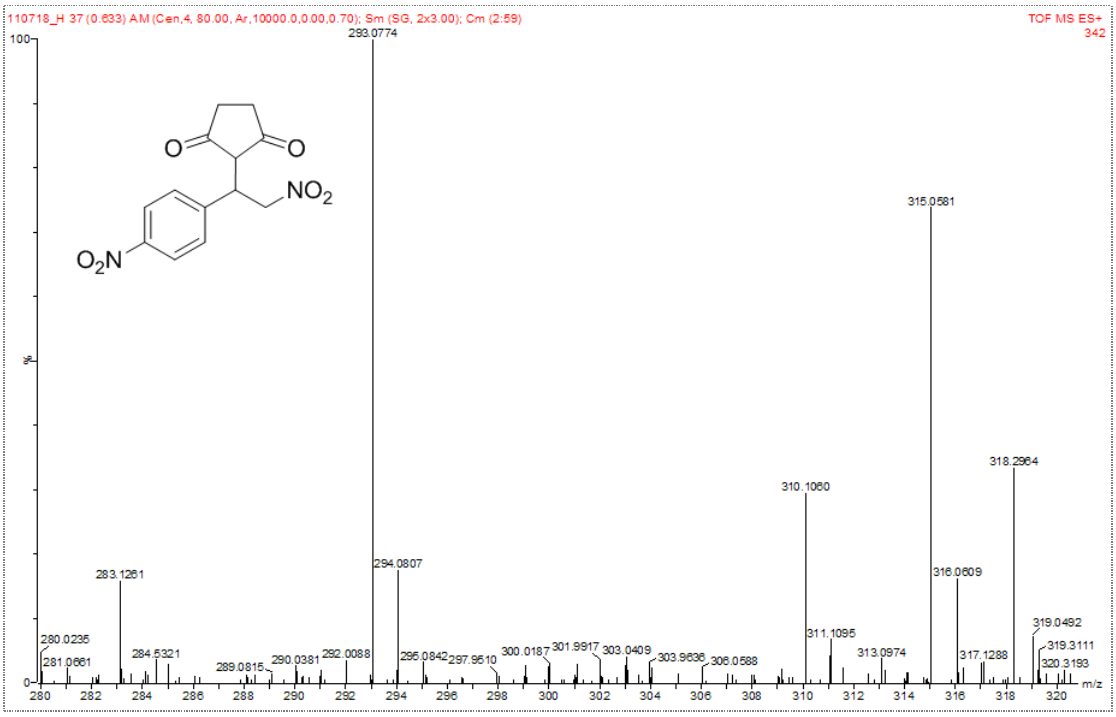
110718_H 37 (0.633) AM (Cen,4, 80.00, Ar,10000.0,0.00,0.70); Sm (SG, 2x3.00); Cm (2:59)
TOF MS ES+
342
293.0774
315.0581
318.2964
310.1060
294.0807
283.1261
316.0609
O
O
NO2
O2N
%
m/z

2-(2-Nitro-1-(2-nitrophenyl)ethyl)cyclopentane-1,3-dione (3i)

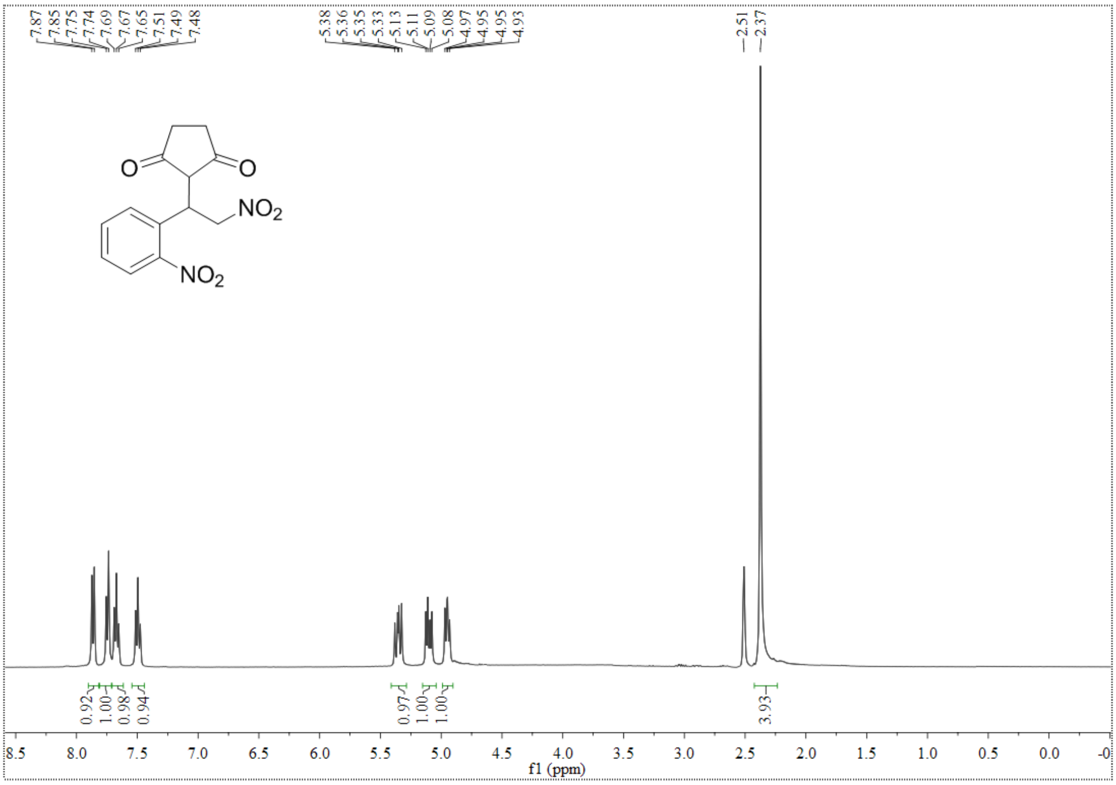

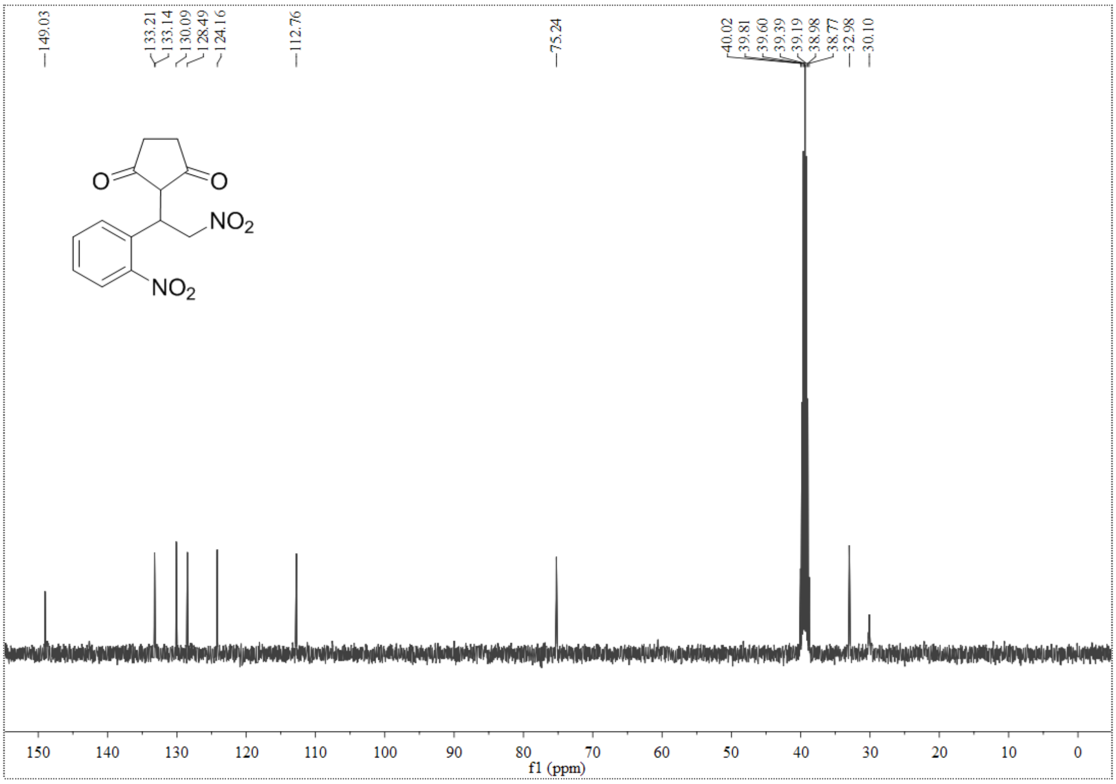

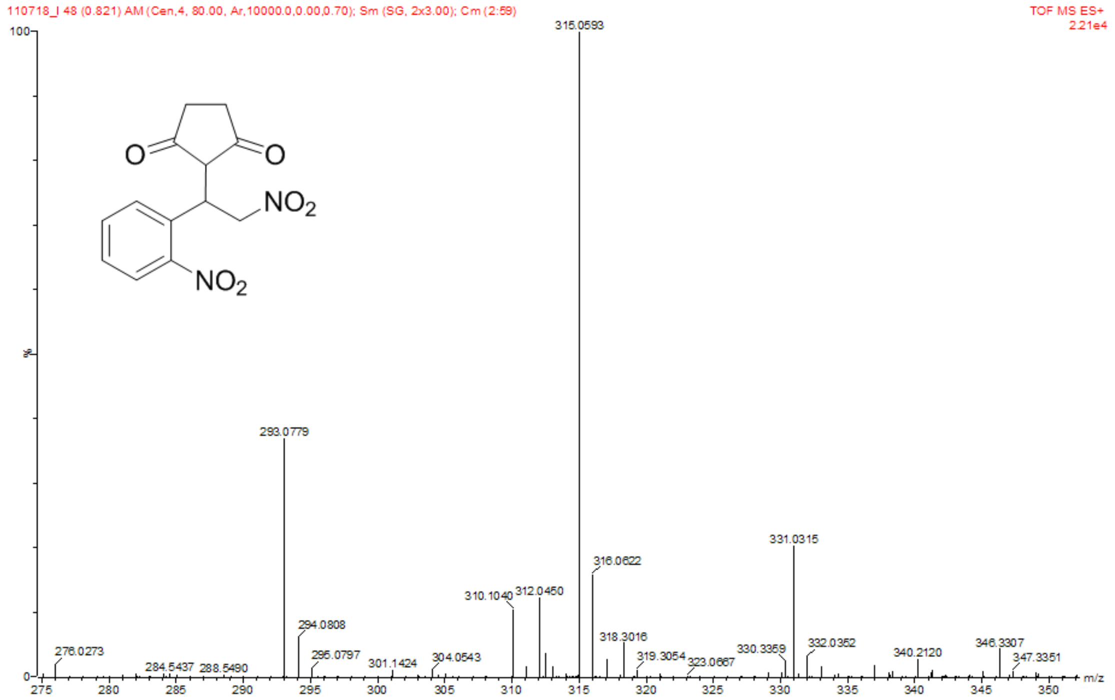

2-(2-Nitro-1-(p-tolyl)ethyl)cyclopentane-1,3-dione (3j)

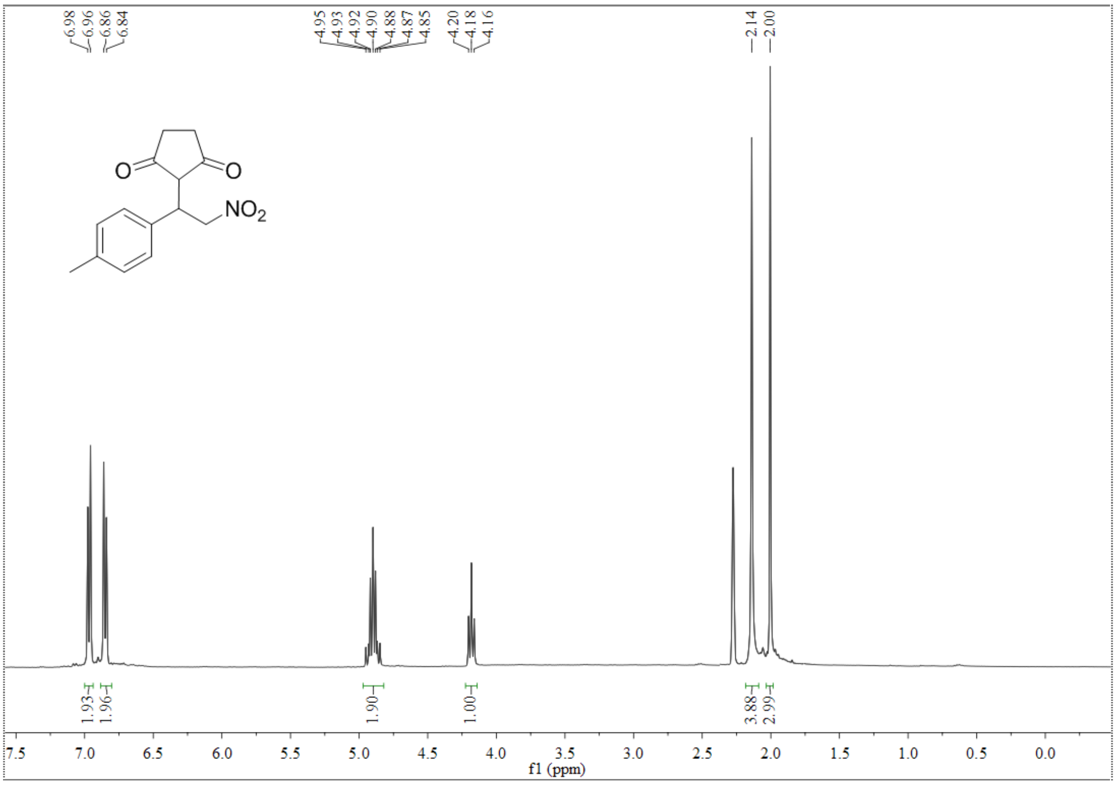

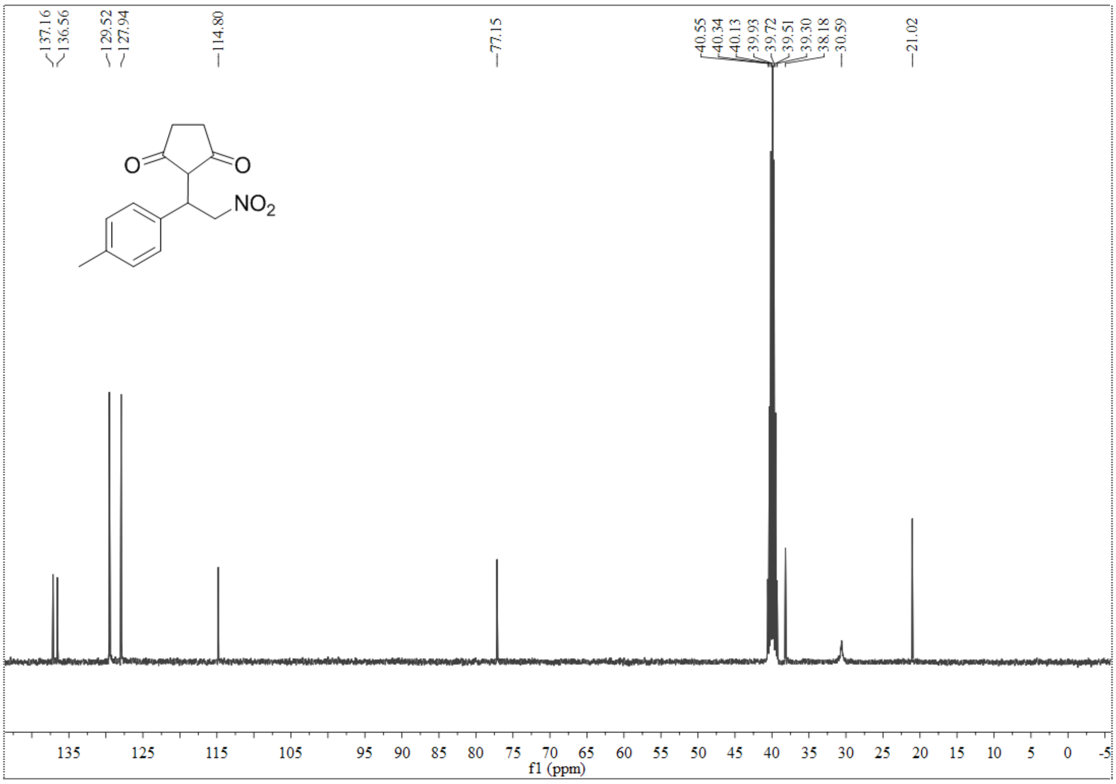
137.16
136.56
129.52
127.94
114.80
77.15
40.55
40.34
40.13
39.93
39.72
39.51
39.30
38.18
30.59
21.02
NO2
O
O
f1 (ppm)

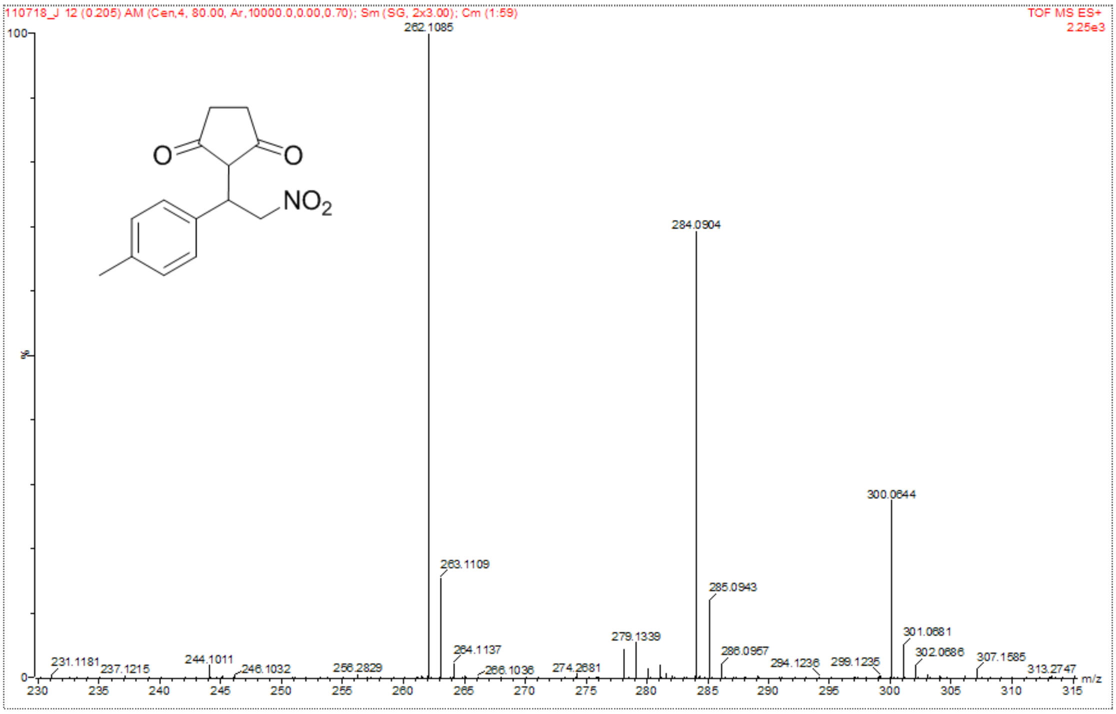
110718_J 12 (0.205) AM (Cen,4, 80.00, Ar,10000.0,0.00,0.70); Sm (SG, 2x3.00); Cm (1:59)
TOF MS ES+
2.25e3
262.1085
284.0904
300.0644
263.1109
285.0943
301.0681
279.1339
264.1137
286.0957
302.0686
307.1585
NO2
O
O
%
m/z

2-(1-(4-Methoxyphenyl)-2-nitroethyl)cyclopentane-1,3-dione (3k)

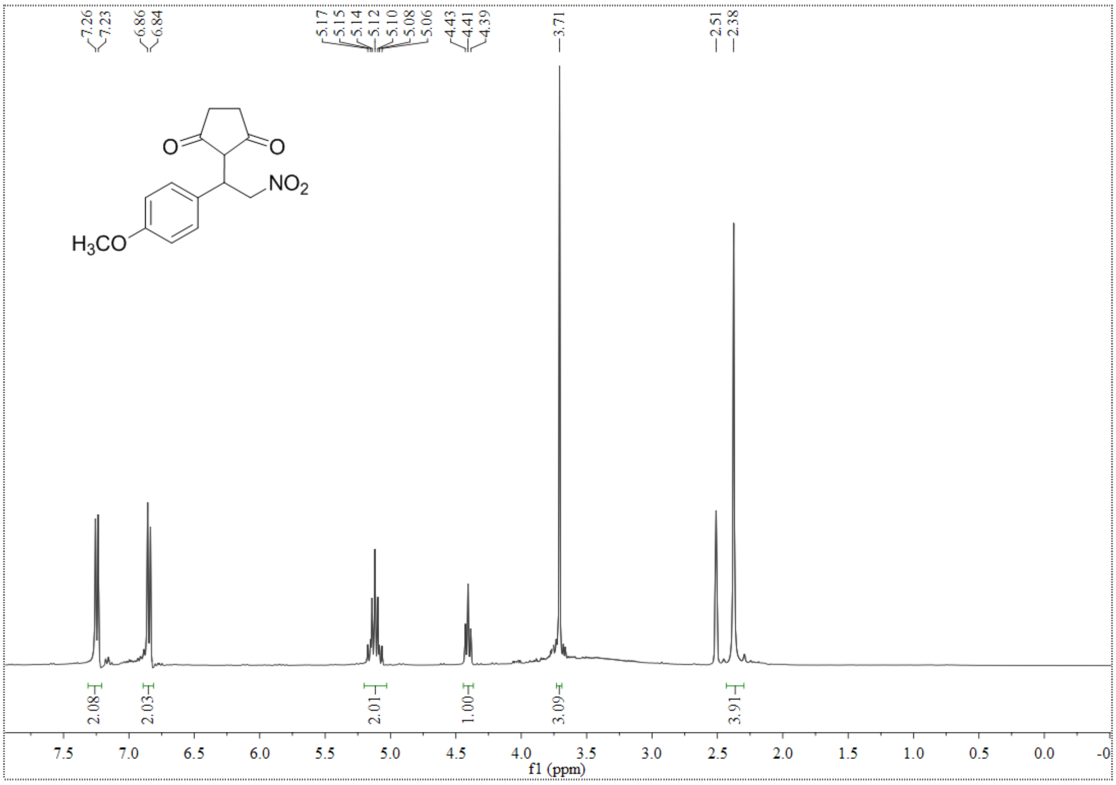

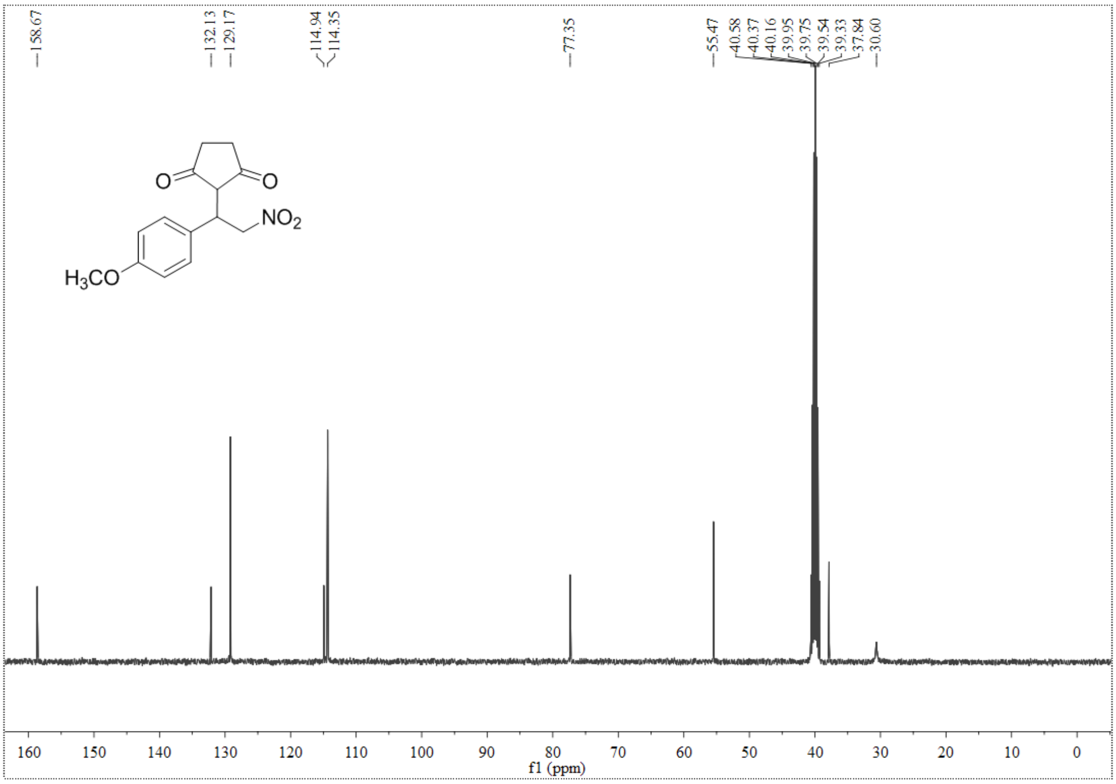

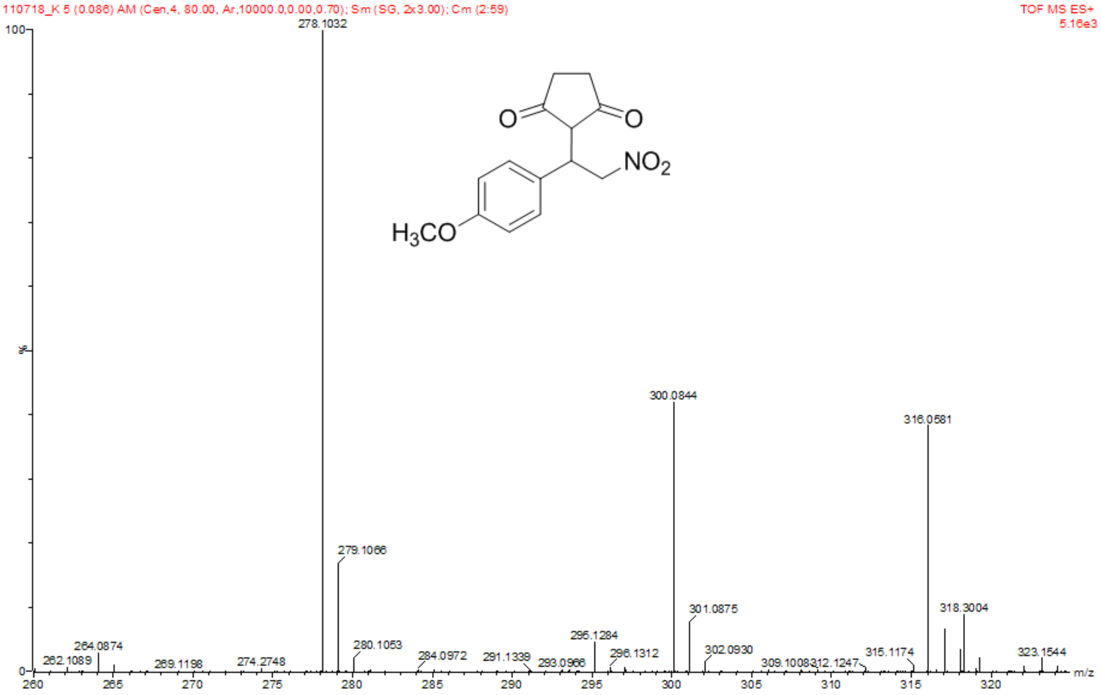

2-(2-Nitro-1-phenylpropyl)cyclopentane-1,3-dione (3l)

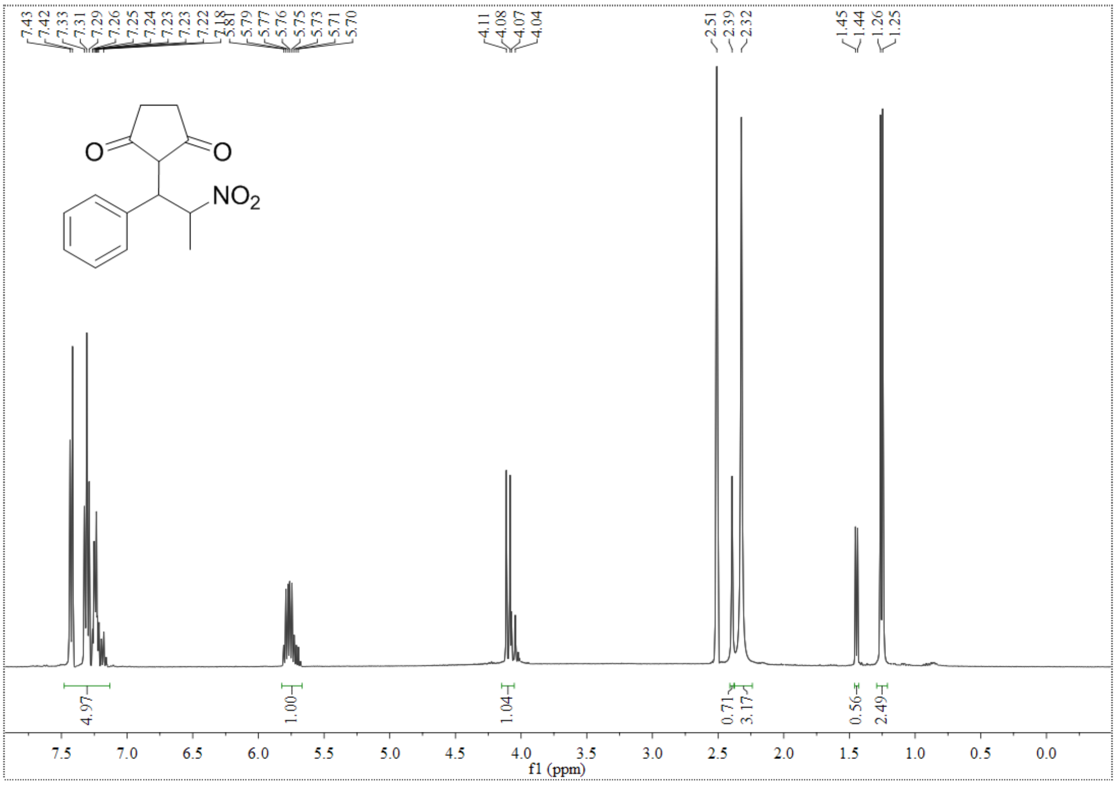

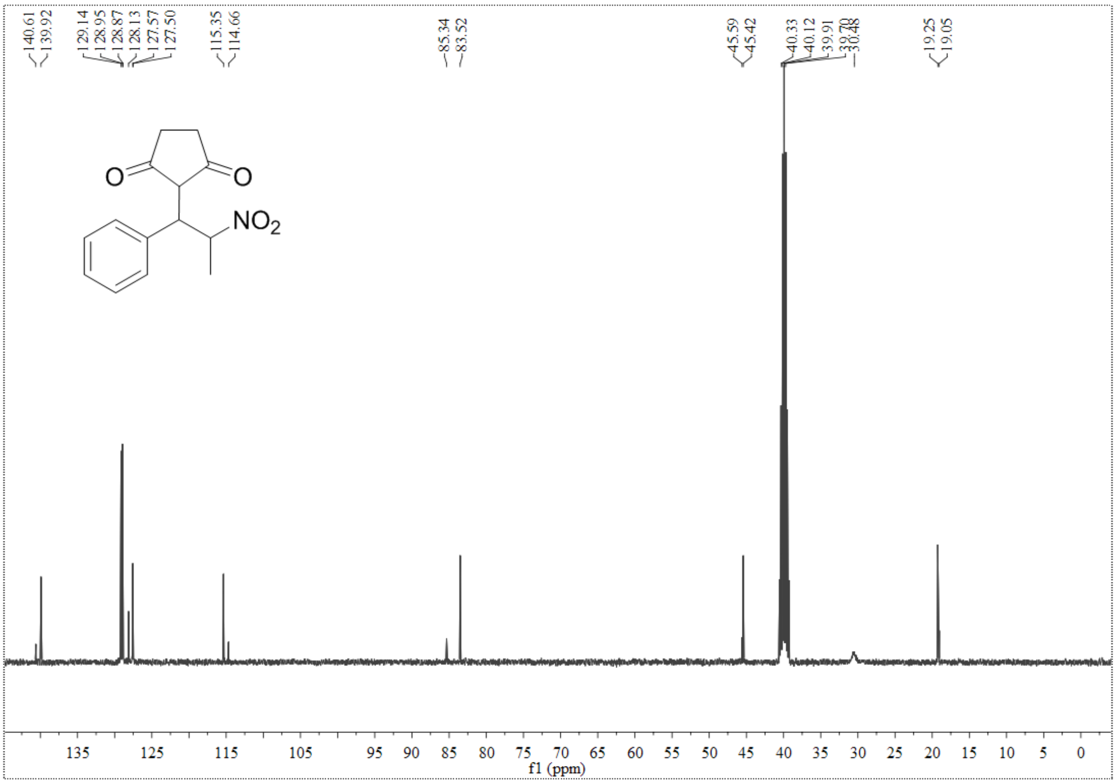
140.61
139.92
129.14
128.95
128.87
128.13
127.57
127.50
115.35
114.66
85.34
83.52
45.59
45.42
40.33
40.12
39.91
39.70
30.48
19.25
19.05
NO_2
O
O
f1 (ppm)

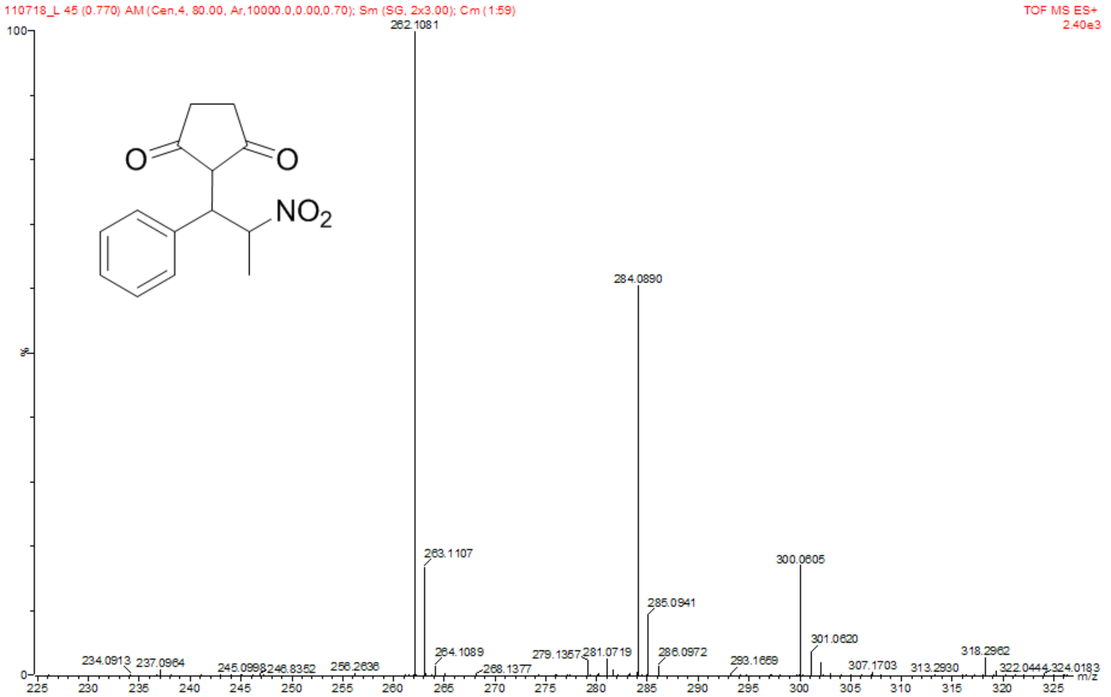
110718_L 45 (0.770) AM (Cen,4, 80.00, Ar,10000.0,0.00,0.70); Sm (SG, 2x3.00); Cm (1:59)
TOF MS ES+
2.40e3
262.1081
284.0890
263.1107
300.0605
285.0941
301.0620
318.2962
NO_2
m/z

2-(1-(Furan-2-yl)-2-nitroethyl)cyclopentane-1,3-dione (3m)

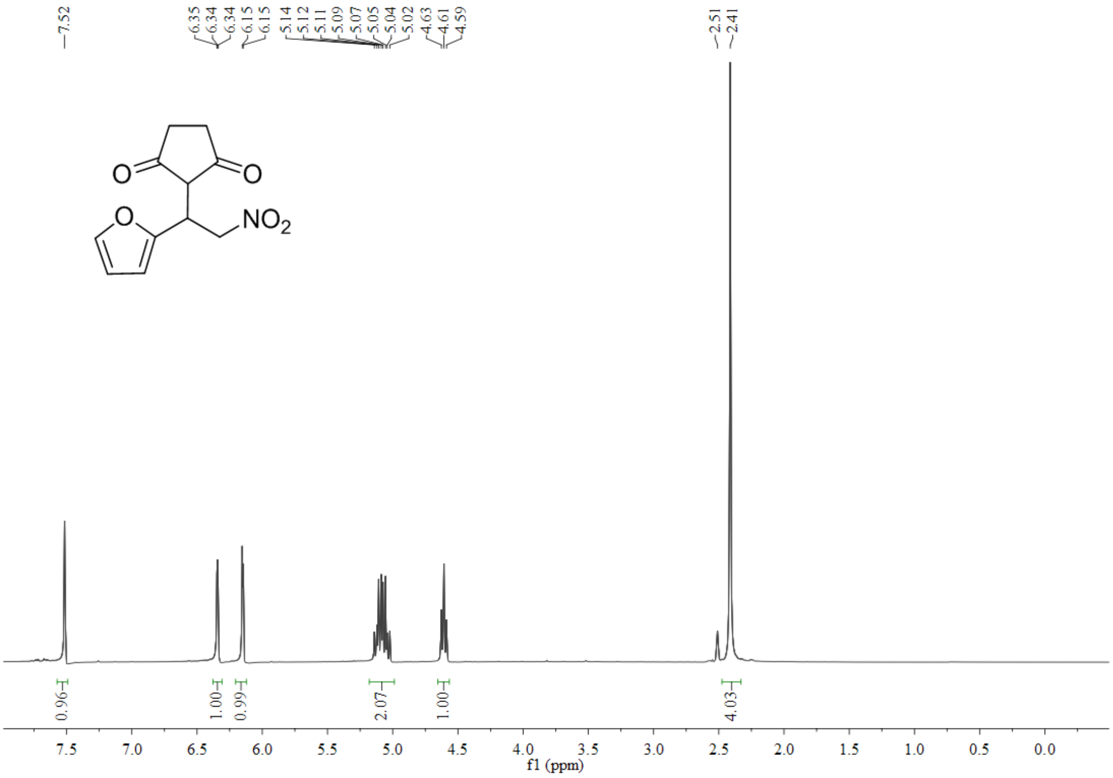

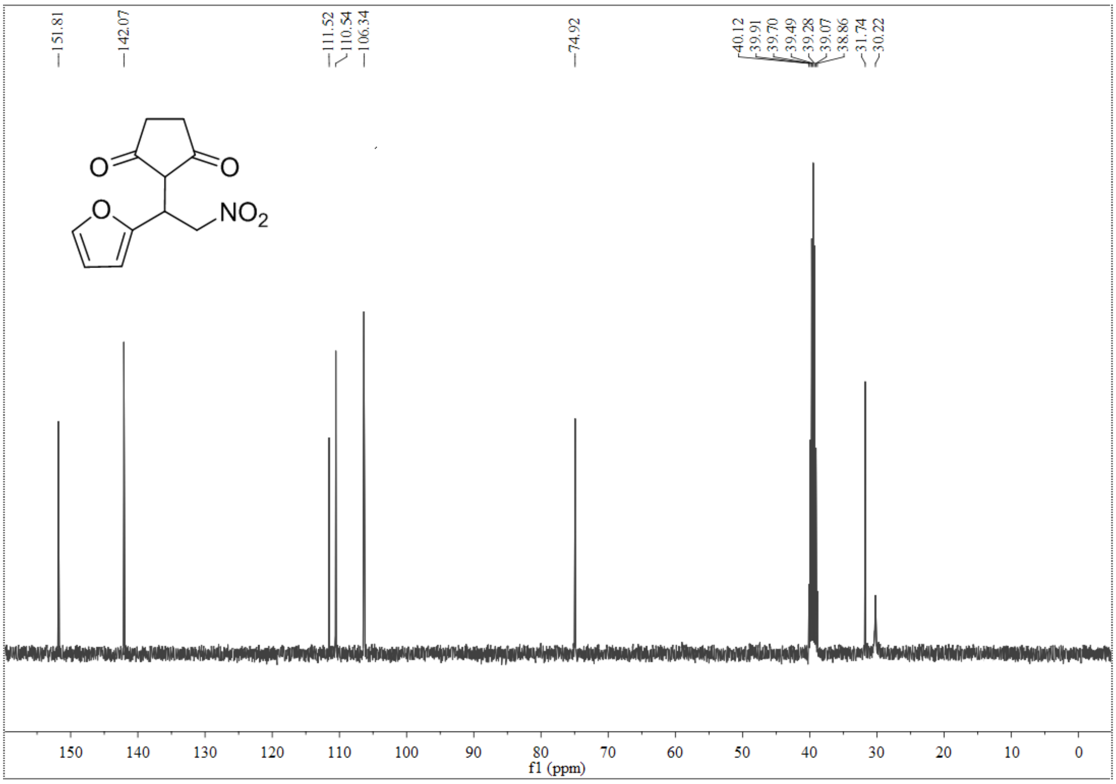

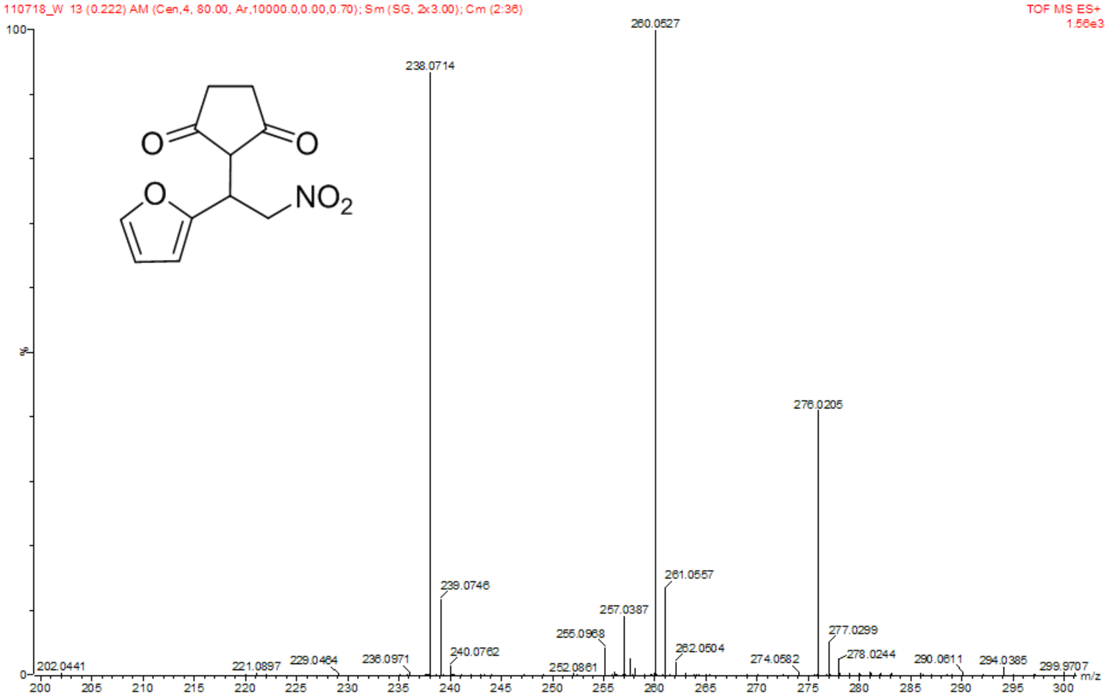

3-(2-Nitro-1-phenylethyl)furan-2,4(3H,5H)-dione (3n)

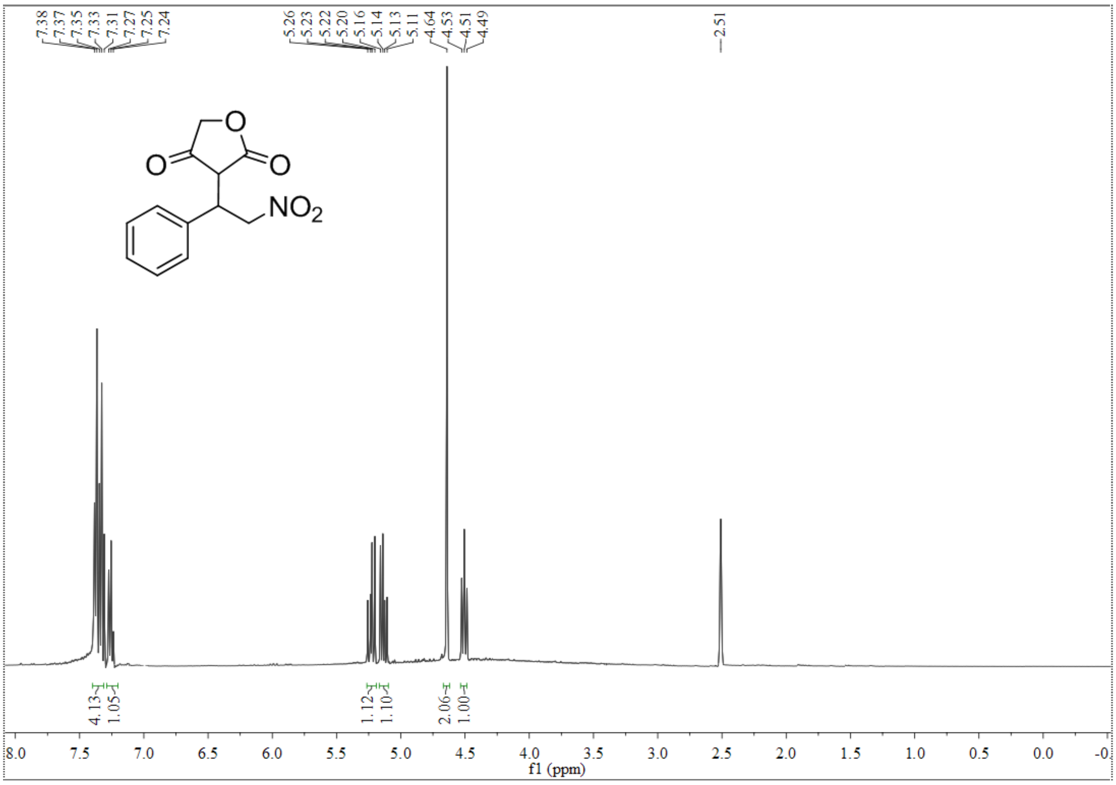

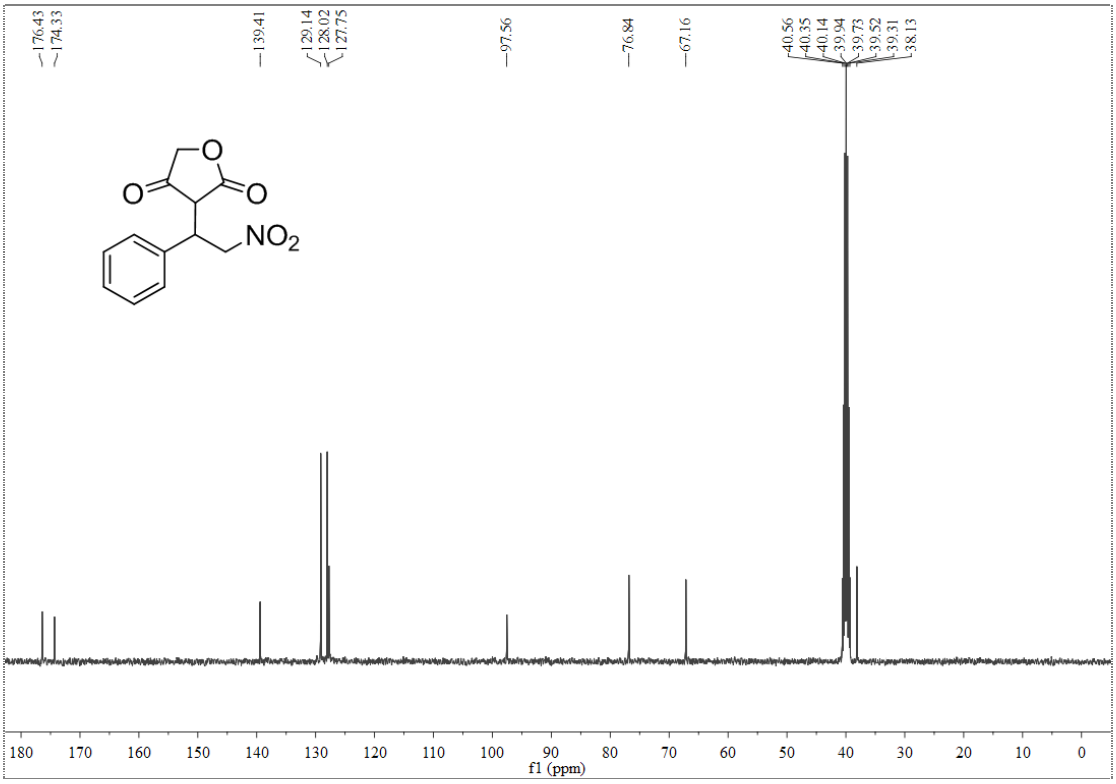

176.43
174.33
139.41
129.14
128.02
127.75
97.56
76.84
67.16
40.56
40.35
40.14
39.94
39.73
39.52
39.31
38.13
NO2
f1 (ppm)

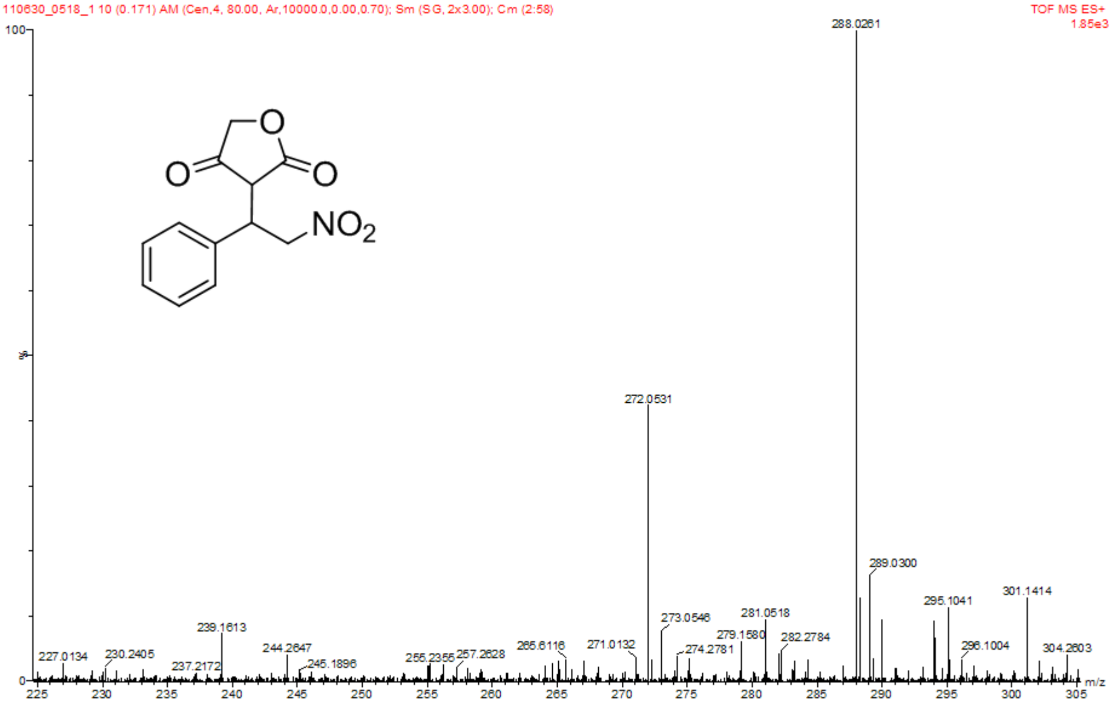

110630_0518_1 10 (0.171) AM (Cen,4, 80.00, Ar,10000.0,0.00,0.70); Sm (SG, 2x3.00); Cm (2:58)
TOF MS ES+
1.85e3
288.0261
272.0531
289.0300
301.1414
295.1041
281.0518
273.0546
279.1580
282.2784
274.2781
296.1004
304.2603
239.1613
244.2647
227.0134
230.2405
237.2172
245.1896
255.2355
257.2628
265.6116
271.0132
NO2
m/z

2-(2-Nitro-1-phenylethyl)-1-phenylbutane-1,3-dione (3o)

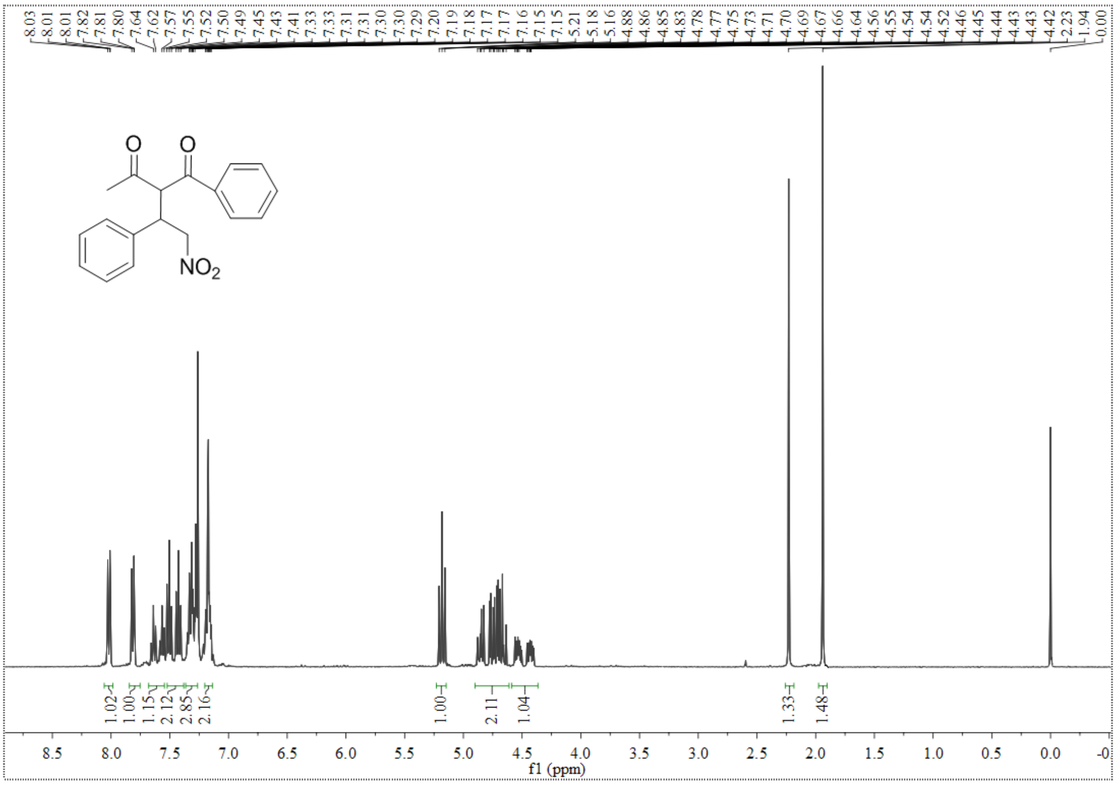

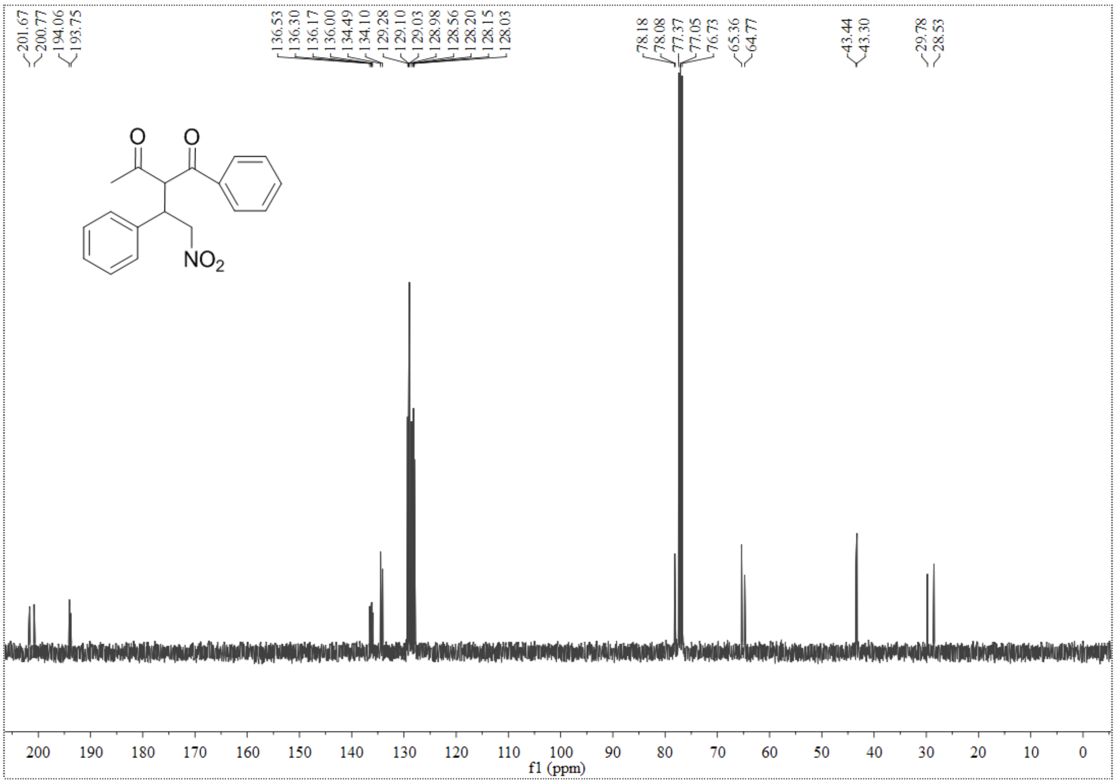

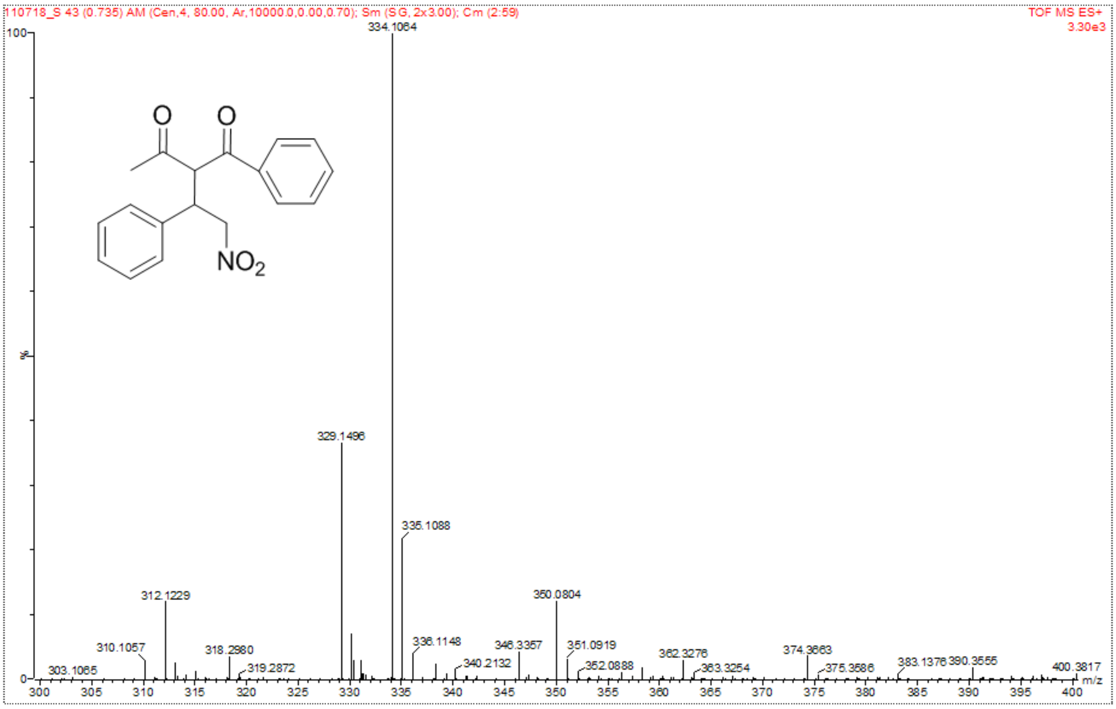

Ethyl 2-benzoyl-4-nitro-3-phenylbutanoate (3p)

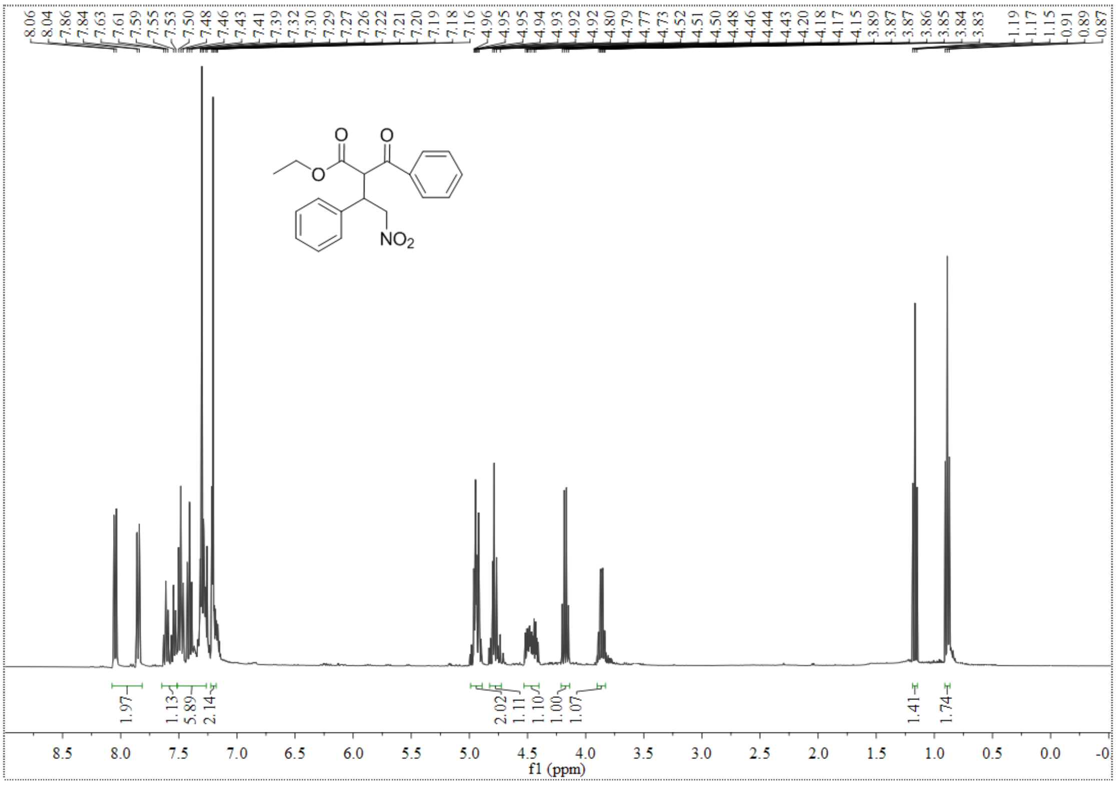

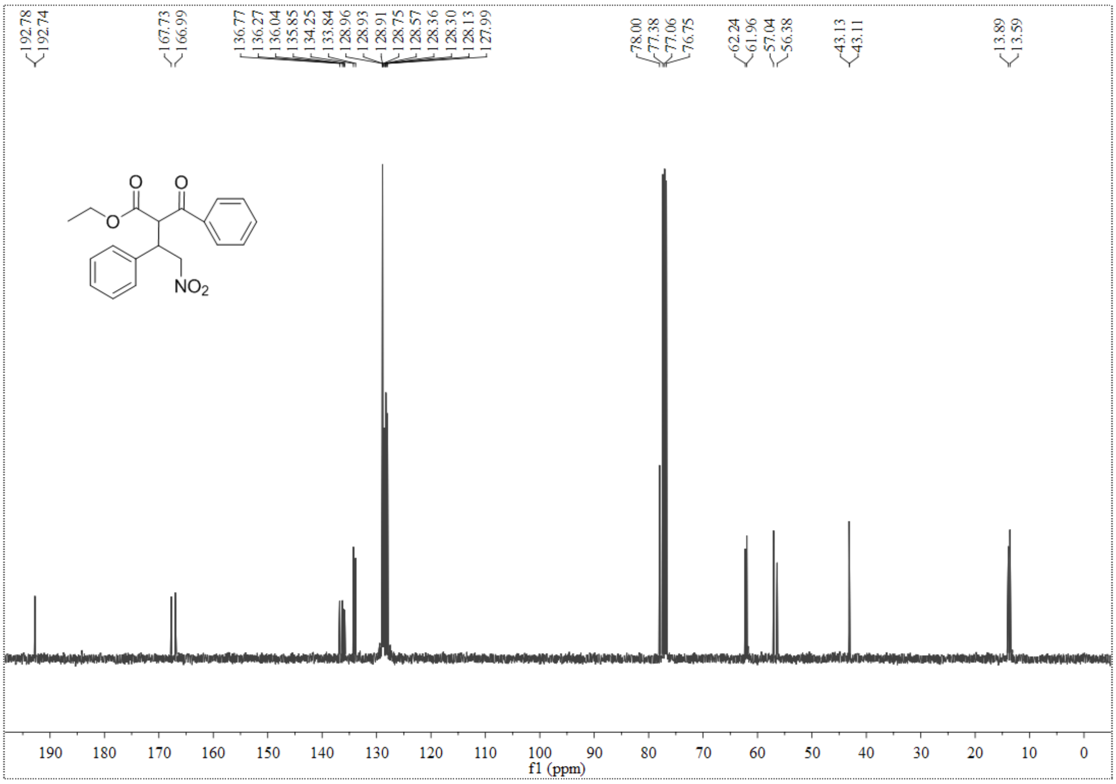
192.78
192.74
167.73
166.99
136.77
136.27
136.04
135.85
134.25
133.84
128.96
128.93
128.91
128.75
128.57
128.36
128.30
128.13
127.99
78.00
77.38
77.06
76.75
62.24
61.96
57.04
56.38
43.13
43.11
13.89
13.59
NO2
f1 (ppm)

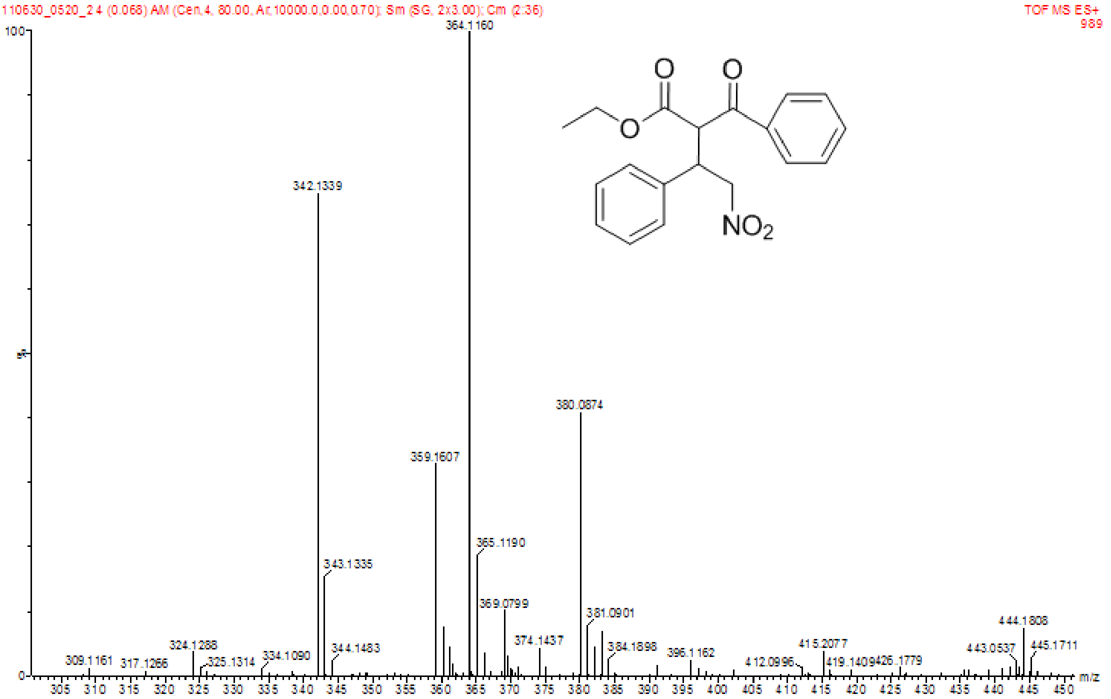
110630_0520_24 (0.068) AM (Cen,4, 80.00, Ar,10000.0,0.00,0.70); Sm (SG, 2x3.00); Cm (2:36)
TOF MS ES+
989
364.1160
342.1339
380.0874
359.1607
365.1190
343.1335
369.0799
381.0901
374.1437
384.1898
396.1162
324.1288
344.1483
334.1090
309.1161
317.1266
325.1314
415.2077
412.0996
419.1409
426.1779
444.1808
443.0537
445.1711
m/z
NO2

Ethyl 2-(4-methoxybenzoyl)-4-nitro-3-phenylbutanoate (3q)

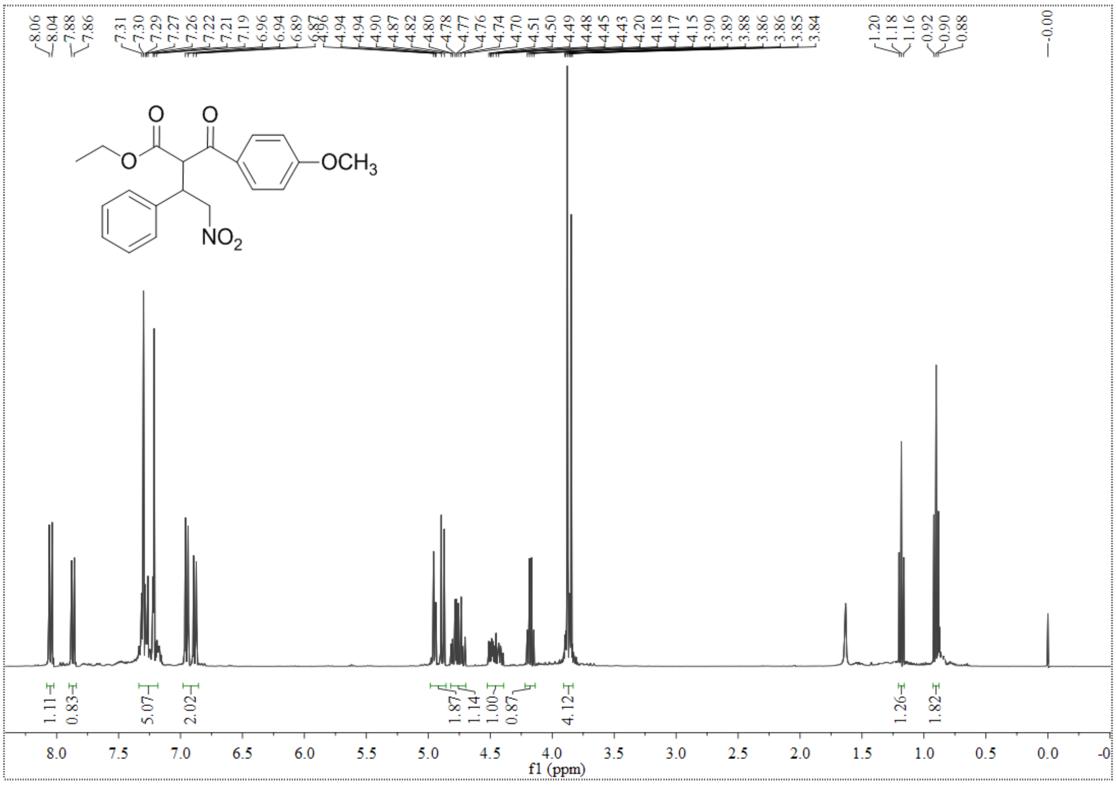

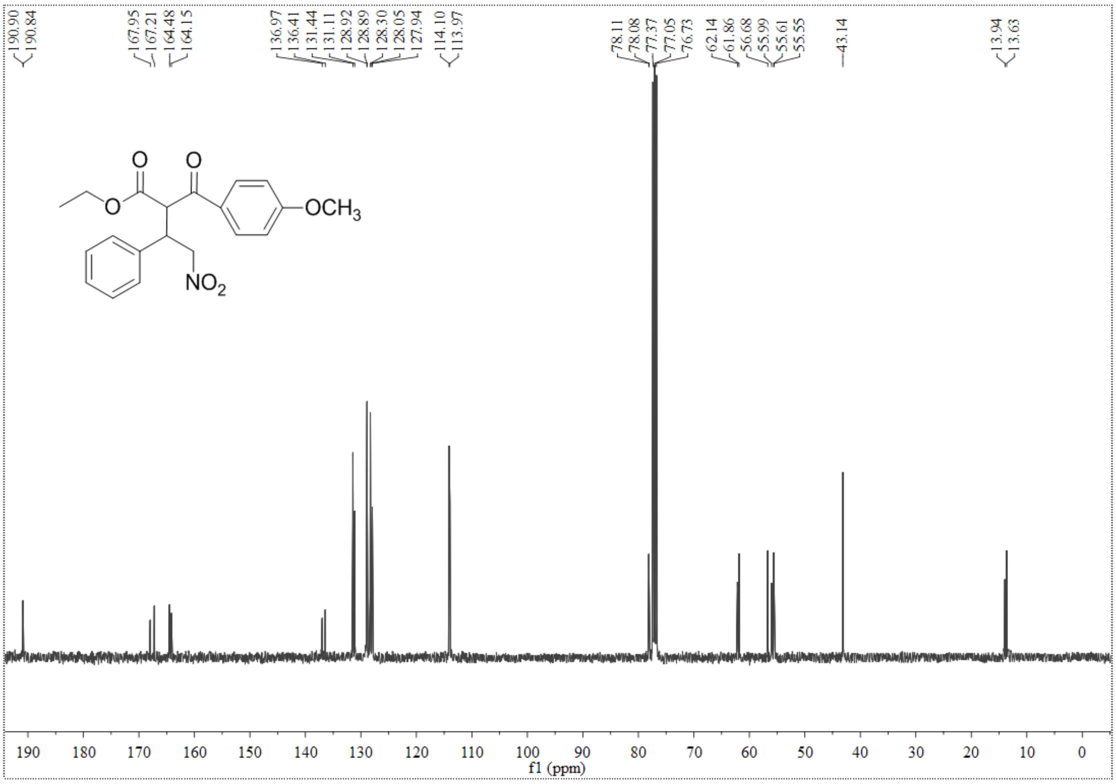

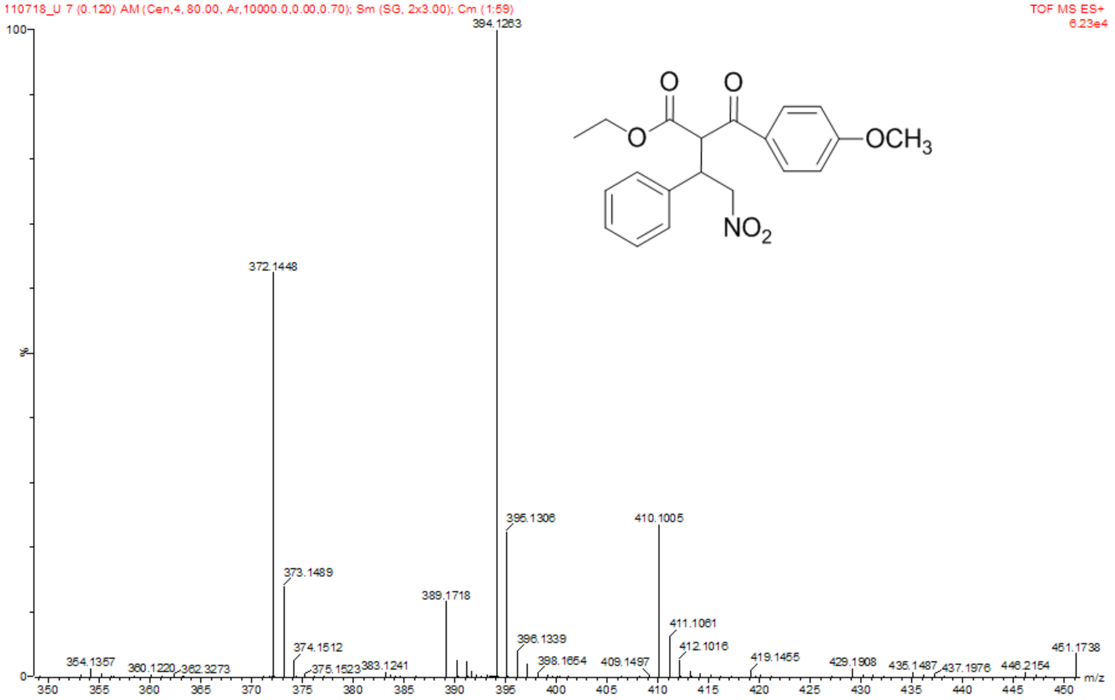

2-(2-Nitro-1-phenylethyl)-1,3-diphenylpropane-1,3-dione (3r)

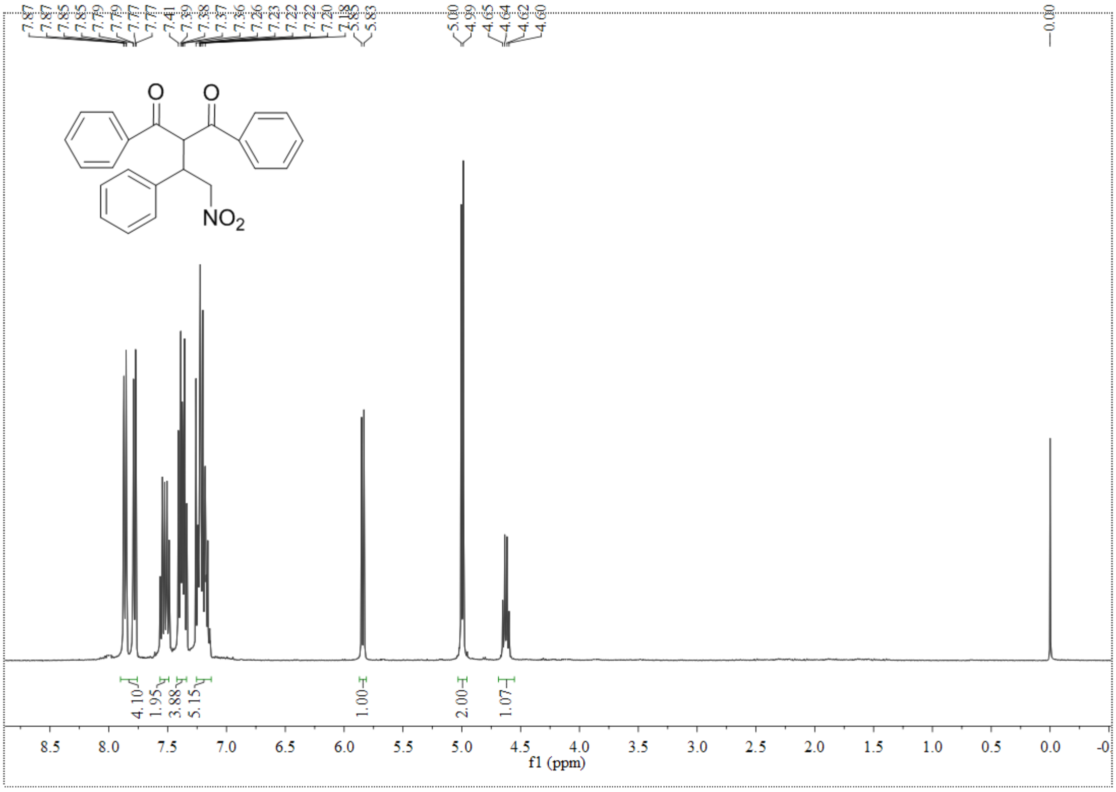

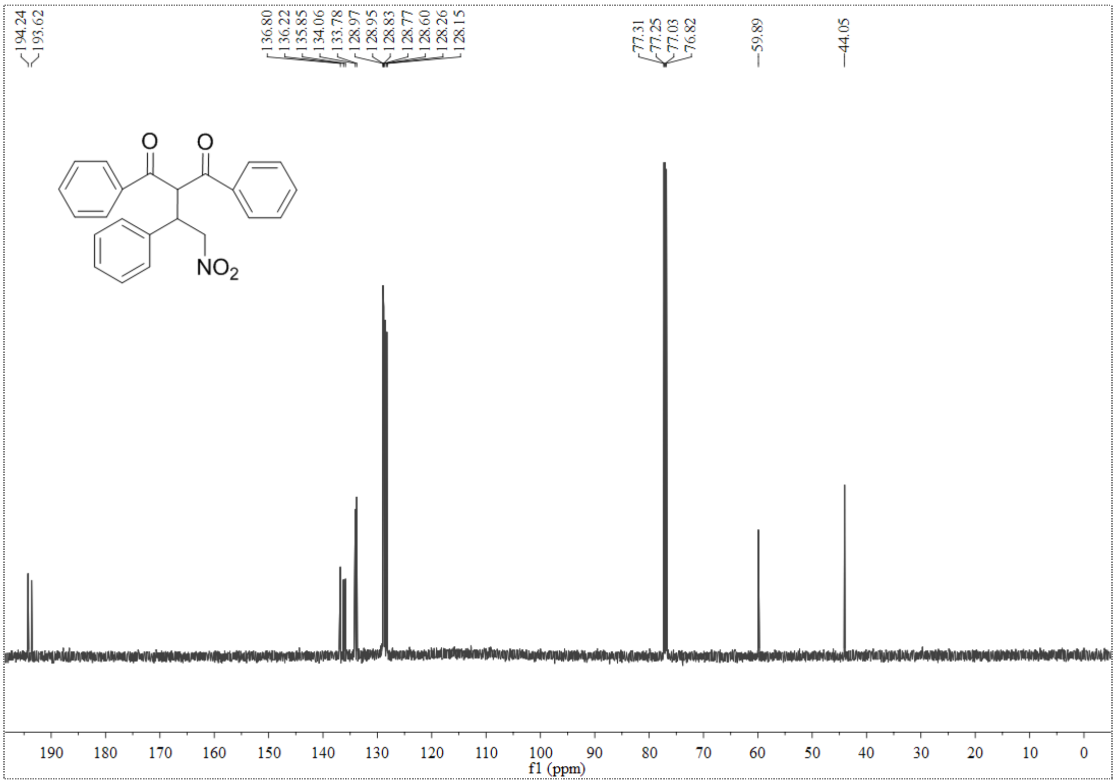
194.24
193.62
136.80
136.22
135.85
134.06
133.78
128.97
128.95
128.83
128.77
128.60
128.26
128.15
77.31
77.25
77.03
76.82
59.89
44.05
O
O
NO2
190 180 170 160 150 140 130 120 110 100 90 80 70 60 50 40 30 20 10 0
f1 (ppm)

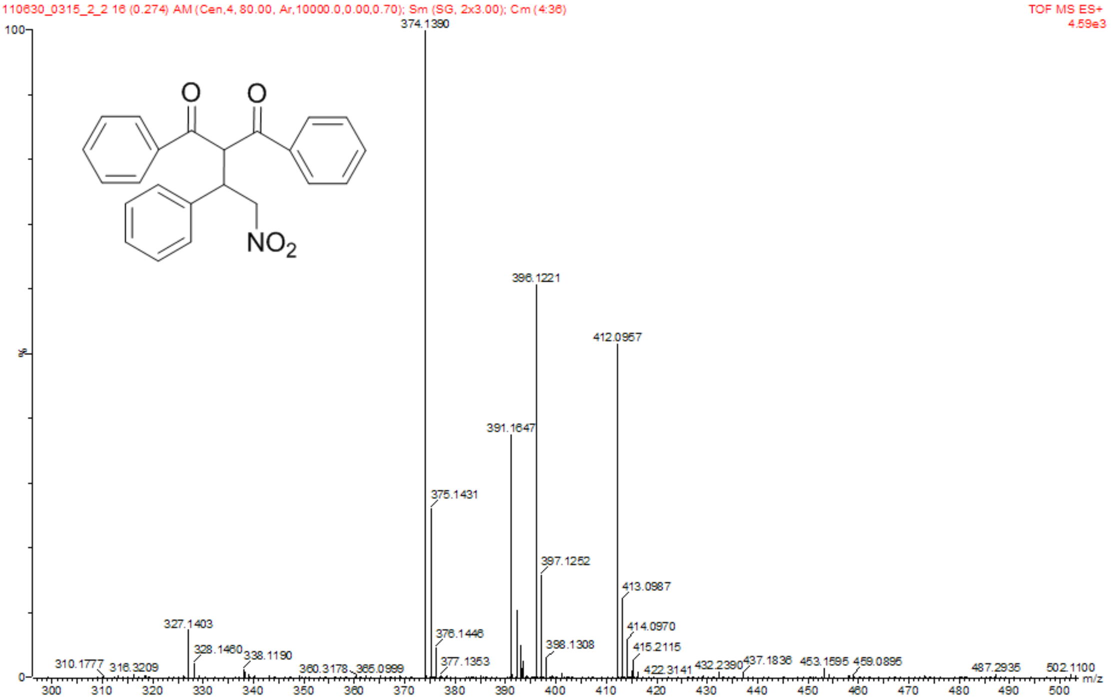
110630_0315_2_2 16 (0.274) AM (Cen,4, 80.00, Ar,10000.0,0.00,0.70); Sm (SG, 2x3.00); Cm (4:36)
TOF MS ES+
4.59e3
374.1390
396.1221
412.0957
391.1647
375.1431
397.1252
413.0987
327.1403
376.1446
414.0970
398.1308
415.2115
328.1460
338.1190
377.1353
310.1777
316.3209
360.3178
365.0999
422.3141
432.2390
437.1836
453.1595
459.0895
487.2935
502.1100
O
O
NO2
%
m/z

3-(1-(Furan-2-yl)-2-nitroethyl)furan-2,4(3H,5H)-dione (3s)

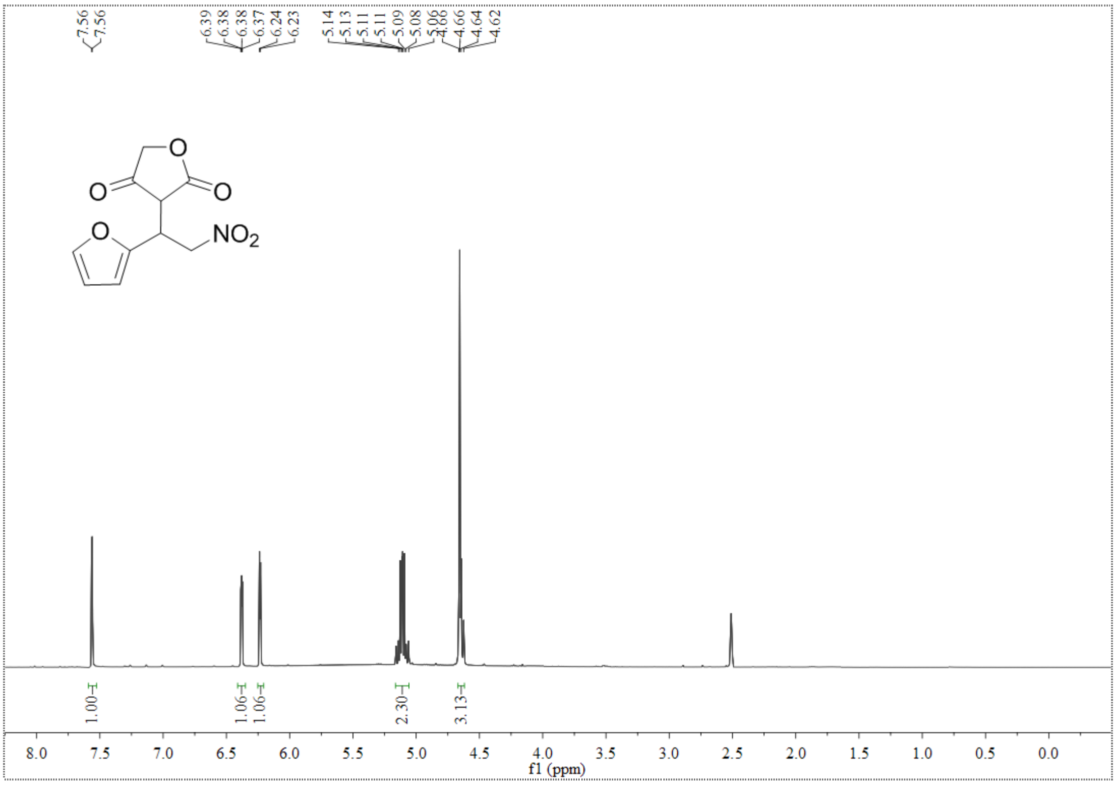

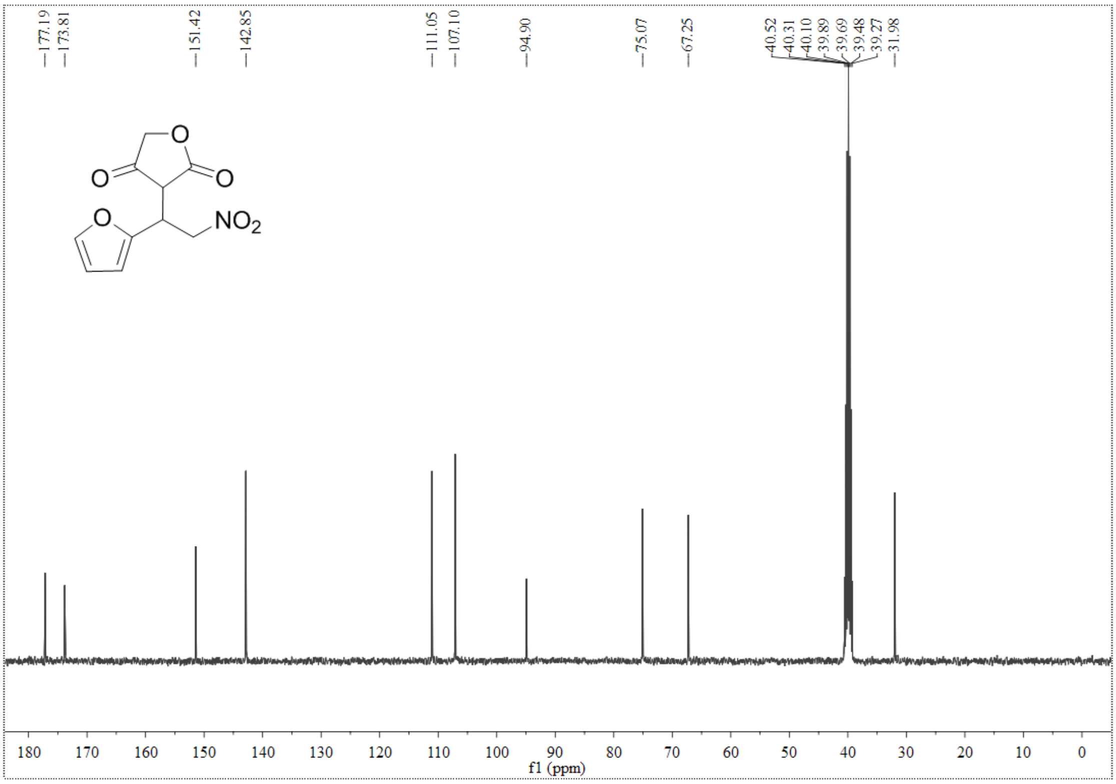

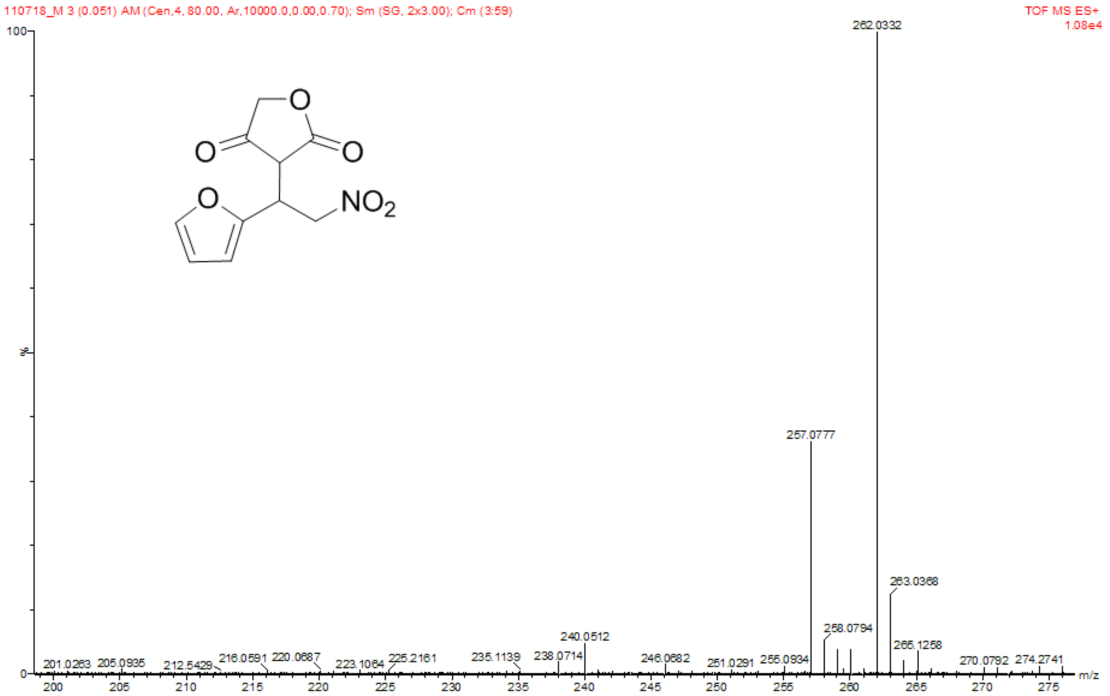
110718_M 3 (0.051) AM (Cen,4, 80.00, Ar,10000.0,0.00,0.70); Sm (SG, 2x3.00); Cm (3:59)
TOF MS ES+
1.08e4
262.0332
257.0777
263.0368
258.0794
240.0512
265.1258
201.0263
205.0935
212.5429
216.0591
220.0687
223.1064
225.2161
235.1139
238.0714
246.0682
251.0291
255.0934
270.0792
274.2741
m/z
%
O
O
O
O
NO2
